# Facile Cobalt-supported Zeolite 4 A as ecofriendly catalyst to prepare 2-Furyl- hydrazine-1-carbothioamides and 1,2,4 Triazole-3-thiones with anticancer investigation

**DOI:** 10.1038/s41598-026-55831-z

**Published:** 2026-06-19

**Authors:** Asiya Farheen, Asia Naz Awan, Abdul Hameed, Syed Fakhar Alam

**Affiliations:** 1 Research Institute of Pharmaceutical Sciences, University of Karachi, Karachi, 75270 Pakistan; 2 Department of Pharmaceutical Chemistry, Faculty of Pharmacy and Pharmaceutical Sciences, University of Karachi, Karachi, 75270 Pakistan; 3 Baqai Institute of Pharmaceutical Sciences, Baqai Medical University, 74600 Karachi, Pakistan; 4 Department of Chemistry, University of Sahiwal, Sahiwal, 57000 Pakistan; 5 HEJ Institute of Chemistry, ICCBS, University of Karachi, Karachi, 75250 Pakistan; 6 Graduate School of Energy Science and Technology, Chungnam National University, Daejeon, South Korea

**Keywords:** Cobalt-Zeolite 4A, Metal supported catalysis, 1,2,4 triazoles, Reusable catalyst, Anticancer activity, Molecular docking, Cancer, Chemistry, Drug discovery

## Abstract

In recent years, crystalline porous zeolite materials have been extensively investigated owing to their distinct characteristics, such as high surface area, pore volume, size, and thermal stability. In this work, 500 nm to 1 μm sized thermally stable cobalt-supported zeolite 4 A was developed via impregnation method, employed as an efficient and reusable catalyst in synthesizing novel thiosemicarbazide and 1,2,4 triazole derivatives using Co-Zeolite 4 A in ethanol: water (1:1) as a green solvent, and found most active among Ni and Fe-supported Zeolite 4 A, with 89% yield. FT-IR, XRD, and BET data revealed characteristic metal-support interactions, crystalline domains, and reduction in surface area (10.1 to 9.4 m^2^/g), confirming successful cobalt incorporation and governing its catalytic activity. The catalyst has maintained its efficacy over five reaction cycles. The anticancer activity was meticulously evaluated with most potent 3,5-bis trifluoromethyl 1,2,4 triazole 4c, displayed IC_50_ 8.74 µM and 29.51 µM against HeLa and PC3 cancer cell lines. In silico docking indicated that compound 4c strongly interacted with the tumor suppressor protein p21, and DFT studies confirmed the chemical reactivity of compounds 3a-3c. This synthetic approach offers high yields, faster reaction times, robust and reusable catalyst, and environmentally sustainable platform for organic transformations with therapeutic potential.

**Supplementary Information:**

The online version contains supplementary material available at https://doi.org/10.1038/s41598-026-55831-z.

## Introduction

Zeolites are crystalline aluminosilicate frameworks with negatively charged macromolecules, that have attracted substantial interest owing to their multi-functionality provided by the high surface area attributable to molecular-sized cavities and intracrystalline channels with distinctive geometry and architecture^1^. It has readily interchangeable, freely moving cations that need to balance the charge of the aluminium-based tetrahedral, has hydrothermal stability, tunable porosity and acidity, compositional diversity, and supports size-shape-selectivity processes^2^. Zeolites are intrinsically microporous, covered a wide range of pore sizes from micropores to macropores depending on textural modifications^3^. Zeolites are known as green and low-waste solid acids comparable to other traditional Brønsted-Lowry and Lewis-acid catalysts^4^. Zeolite 4 A, having a pore size 0.4 nm, excellent adsorption and ion exchange properties, used in versatile chemical synthesis applications such as catalyst carriers, and immobilization processes^5,6^. They have been employed in several catalytic organic conversions, including fragmentation^7^, isomerization^8^, hydrocarbon synthesis^9^, alkylation^10^, and selective oxidation^11^, making it a suitable support material for anchoring metals. The functional catalytic material is necessary to provide not only the active sites, but also a large surface area and enhanced mechanical strength in subsequent catalytic transformations.

It has been reported that various metal-supported zeolites have demonstrated catalytic behavior in bond-forming reactions of carbon–carbon and carbon–heteroatom^12^. The carbon-nitrogen linkage activation is sustainable and effective approach in organic reactions, particularly useful in synthesis of heterocyclic chemistry, particularly in the activation of carbon-based electrophiles, followed by enhanced polarization, which promotes the C-N sp^2^-bond activation under mild reaction conditions. N-heterocyclization in indole^13^, coupling reaction^14^, unsymmetric Hantzsch condensation^12^, glycosidation^15^ reaction has been catalyzed using zeolite-supported palladium, copper, and lanthanum catalyst^16,17^.

Among various transition metals, cobalt is relatively abundant, eco-friendly, and has a versatile catalytic function as a nucleophilic and electrophilic center for activation of substrates, formation of well-defined coordination complexes with heterocycles, and tunable active sites^18^. Among transition metals like Fe, Ni, Zn, and Cu, the distinguished catalytic activity of cobalt is primarily attributable to its partially occupied d-orbital (3d^7^)^19^. Cobalt favors balanced activity, stability, and selectivity in organic reactions due to its intermediate electronic structure^20^. The existence of cobalt in oxidation states of Co^2+^ and Co^3+^ allows it to interact with other elements and support^19^. The growing interest in green chemistry criteria, over the past two decades, presents numerous constraints, including the polymerization of complexes and the aggregation of cobalt species in homogeneous catalysis, their outrageous cost, and scalability. The supported catalysts have also been found to be superior, owing to reduced sintering and improved catalytic performance^21^. The isolated Co^2+^ supported zeolite framework is relatively stable and behaves as Lewis-acid sites comparable to cobalt oxides^22^. The generation of isolated Co-species, Co-oxides clusters, and Co-nanoparticles is primarily based on the preparation method, cobalt precursor, and cobalt loading. Well-dispersed cobalt-based aluminum having strong support basicity is crucial for providing a stable and efficient catalyst facilitate nucleophilic reaction, with selectivity and least metal-metal interaction^23^. The synergistic metal-support interaction has emerged as powerful synthetic tool for highly functionalized compounds.

**Fig. 1:**
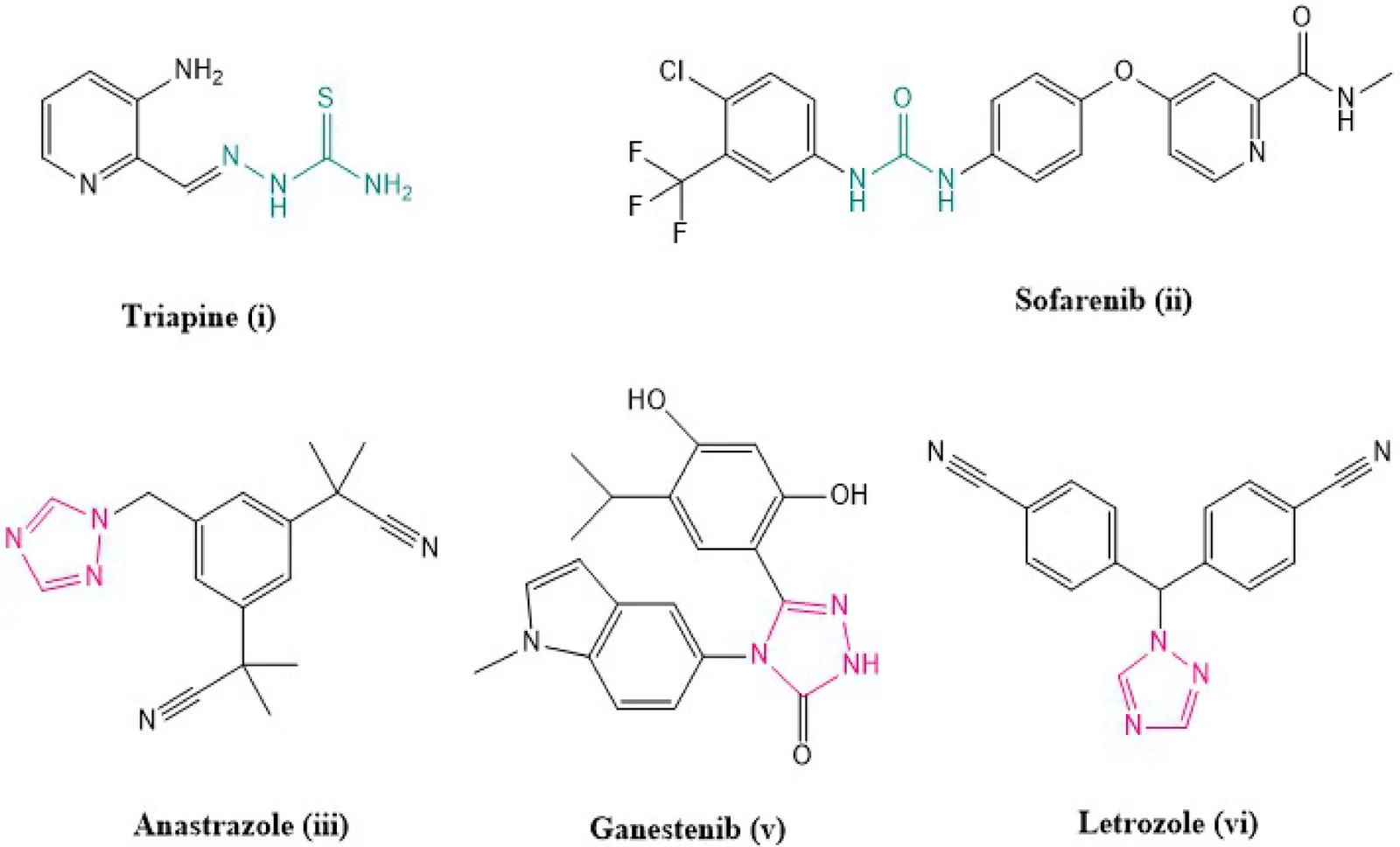
Structure of some bioactive thiosemicarbazides and 1,2,4 triazole scaffolds.

Heterocycles are the important scaffolds, which have garnered extensive scientific interest owing to their functionality in material chemistry agrochemical and biological applications. Particularly, those possessing both nitrogen and sulfur scaffolds are inferred to have particular relevance in drug discovery endeavors in medicinal chemistry^24,25^. Recent advances in the synthesis and pharmacological action of two classes of azo-heterocycles viz. thiosemicarbazides and 1,2,4 triazole is well-documented in current literature^26,27^. The therapeutic response and structural resilience ascribed to their scaffold stem from the –N–C = S– pharmacophore confined in molecular architecture. Furyl-based heterocycles have better physicochemical and biological relevance comparable to other aryl and alkyl substituents attributable to their high reactivity, less steric hindrance, improved target binding, and membrane permeation^28^. The introduction of trifluoromethyl substituents in heterocycles has been a well-documented approach in medicinal chemistry, enabled by enhanced metabolic stability through enzyme degradation resistance, improved lipophilicity and targeted binding interaction^29^. Thiosemicarbazide are convenient precursor, polyfunctional moieties possessing nucleophilic properties that have been utilized to design various heterocyclic compounds with different ring sizes, such as pyrazole^30^, thiazole, thiadiazole^31^, triazole^32^ and triazine^33^, depending on the nature of the substituent, pH of medium, solvent and temperature conditions. 1,2,4 triazole (C_2_N_3_H_3_) chemistry and their derivatives, are consistently highlighted attributable to their propensity in interaction with particular cellular targets and pathways, leading to remarkable pharmacological and biofunctional characteristics such as antifungal^34^, antiviral^35^, anticancer^36^, antibacterial^37^, analgesic^38^, anti-inflammatory^38^, antiurease^39^, antioxidant^39^, antihistamine^40^, anti-obesity^41^, antideppresant^42^ and antialzhiemer^43^ agents. Triazole scaffolds exhibit a distinct structure facilitated to favorably interact with a range of receptors and enzymes in physiological pathways via weak interactions, including van der Waals forces, coordination, hydrogen bonding, ion–dipole, hydrophobic, π-cation, π-π stacking interactions, and more^44^. Thiosemicarbazide derivatives and 1,2,4 triazole have been reported as solid antitumor agents targeting cervical (HeLa) and prostate (PC3) cancer cell lines by Imtiaz et al.^45,46^. Bei Zhang et al. explored furan-based triazole with potent antiproliferative action against PC3, A549, and MCF-7^47^. X. Ouyang et al. implicated the studies on a set of 1,2,4-triazole derivatives inhibiting cell cycle progression of various cancer cell lines. Many ring systems containing thiosemicarbazides and 1,2,4 triazoles are integrated pharmacophores in diverse range of recognized and approved chemotherapeutic agents, such as sorafenib, triapine, ganetesbip, vorozole, anastrazole, and letrozole (Fig. 1) proved advantageous as anticancer agents^48,49^. These were studied as many enzyme-mediated and distinct oncogenic targets, such as ganetesbip is investigational drug being explored for prostate and breast cancer as heat shock protein 90 inhibitor (HSP 90) and sorafenib is multikinase target inhibitor having potent antitumor activity against HeLa cell line^49,50^.

In the view of such biological significance of furan based thiosemicarbazides and 1,2,4 triazole scaffolds, their synthetic diversification enables the production of various derivatives, including 1,2,4 triazole analogues resulting from copper catalyzed sequential oxidative coupling reactions for N-C and N-N bond-formation with reaction time exceeded 24 h in the presence of DMSO as solvent, restricting its sustainable application^51^. G. M. Castanedo reported region-selective process of hydrazines, amidines and carboxylic acid, DMF as solvent, and longer reaction time of 18 h, which limited its efficacy and safety^52^. Several other synthetic routes, i.e., dipolar cycloaddition^53^, base-mediated cycloaddition^53^ and copper and silver catalyzed synthesis for substituted triazoles by decarboxylation and cyclization of 2-aryl-2-isocyanate^54^ are limited by either highly-priced toxic solvent, and prolonged reaction durations. The advent of benign conditions in the synthesis of aza-heterocycles represents a critical stride towards a more sustainable future, where the design of novel and potent compounds aligns with the objective of mitigating the environmental impact of chemical processes. This approach features a judicious preference of reagents, solvents, catalysts and activation methods.

A proficient procedure of Co-Zeolite 4 A catalyzed reaction is depicted for the developing novel furan- based thiosemicarbazides, followed by the base-induced cyclization, thereby introducing strategically oriented electron-withdrawing groups in 1,2,4 triazole scaffolds, which to our knowledge not highlighted previously in literature. The prepared scaffolds were evaluated for vitro inhibition of tumor cell proliferation on HeLa and PC3 cancer cell lines, SAR evaluation and in-silico docking studies targeting, p21 and p53 proteins for elucidating the molecular-level interactions of these derivatives with essential anticancer targets. Additionally, in-silico ADME analysis was executed to evaluate several theoretical pharmacokinetic and toxicological parameters, demonstrating the potency and tolerability of the synthesized derivatives as anticancer drugs.

## Experimental

### General

All reagents with analytical grade were commercially procured and used without undergoing additional purification. Scanning electron microscopy (SEM) technique and energy-dispersive X-ray (EDX) with 987 cps average count rate and 30 kV acceleration voltage were used for determining surface morphology, distribution and elemental composition of sample. Surface area measurements were carried out using the BET method with nitrogen adsorption at − 195.85 °C. X-ray diffraction (XRD) with tube current 25 mA, voltage 40 kV, step size 0.036, scan time per step 21.2 s and 2 θ range of 5°−80° were employed to confirm metal incorporation and preservation of the zeolitic structure. The thermal gravimetric analysis/differential scanning calorimetry (TGA/DSC) for the catalyst was conducted in the heating range of 70–800 °C and 10 °C min^− 1^ of uniform heating rate under nitrogen atmosphere to evaluate the thermal properties of sample. The reaction was monitored by thin layer chromatography (TLC) on MERK silica gel plates and spot visualization was performed under UV lamp at 254 nm utilizing ethyl acetate: n-hexane (7:3) as a mobile phase. The melting points of prepared compounds were evaluated by Stuart melting point apparatus (SMP 30) by capillary method and were not adjusted. FT-IR spectra were recorded on Shimadzu spectrophotometer from 4000 to 500 cm^− 1^. The H^1^ and C^13^ NMR characterization were conducted on Bucker instruments using methanol-d_4_ and DMSO-d_6_ as solvent, employing TMS as standard for evaluating resonance shifts in δ ppm. Mass spectral analysis (EI^+^) were evaluated by JEOL JMS-600 H spectrometer. All cell lines were procured from commercial ATCC sources.

### Preparation of the Co-Zeolite 4 A catalyst

The Co-Zeolite 4 A catalyst was prepared by an impregnation method (1:5 ratio) by stirring suspension of 0.24 g cobalt (II) chloride hexahydrate (1 mmol) and 1 g of 4 A molecular sieves in 250 ml flask containing deionized water at room temperature (25 °C) for 24 h. Filter and washed the solid thoroughly with 50 ml deionized water and 20 ml acetone, and dried for 1 h at 150 °C in an oven. The catalyst was preheated at 120 °C for 1 h before use in model reaction.

### General procedure of preparing thiosemicarbazide derivatives 3a-c

The mixture of 2-Furoic acid hydrazide (0.1 mmol) and phenyl isothiocyanate analogues (0.1 mmol) was combined with Co-Zeolite 4 A (10 mg) in 10 ml of an ethanol-water (1:1) and the reaction mixture was refluxed at 40 °C. Following the reaction completion, the catalyst was separated by filtration, washed with ethanol, and dried for reuse. The filtrate was concentrated by adding cold distilled water and the precipitate was then washed with water and recrystallized from ethanol.

### General procedure for synthesizing 1,2,4 triazole derivatives 4a-c

A mixture of compounds 3a–c (0.45 mmol) was added to 2 N NaOH solution (10 ml), stirred and refluxed at 40 °C for 4 h. The reaction was monitored by TLC using methanol as mobile phase. The resulting solution was filtered, cooled at room temperature, and the pH was adjusted to 5.0–6.0 with an aqueous solution of HCl (0.1 N, 5 ml). The resulting precipitates were filtered, dried, washed with water, and recrystallized from ethanol.

## In vitro study

### Cell line and cell culturing

Human cervical cancer cell line (HeLa) RRID: CVCL_0030 (CCL-2) and Human prostate cancer (PC3) RRID: CVCL_0035 (CRL-1435) were cultured under standard protocol. HeLa cells were grown in minimum essential medium (MEM), and PC3 cells were maintained in Dulbecco’s Modified Eagle Medium (DMEM). Each cell line was supplemented with 5% fetal bovine serum (FBS), penicillin (100 IU/mL), and streptomycin (100 µg/mL) in 75 cm ^2^ flasks and kept in 5% CO_2_ incubator at 37 °C.

### Anticancer activity

The anticancer potential of all six synthesized compounds was examined by the MTT (3- [4, 5-dimethylthiazole-2-yl]−2, 5-diphenyl-tetrazo-lium bromide) colorimetric assay performed in 96-well flat-bottomed microplates against HeLa and PC3 cancer cell lines. For this purpose, exponentially growing cells were collected and counted using a haemocytometer and diluted with a specific medium. The cultured cells with a concentration of 6 × 10^4^ cells/ml were prepared and added (100 µL/well) into 96-well plates and allowed to incubate overnight. After incubation, the medium was removed, and 200 µL fresh medium was introduced with different concentrations of drug samples (1–30 µM) to adherent cells for 48 h. MTT solution of 0.5 mg/mL in 200 µL was added to each well and kept for an incubation period of 5 h. 100 µL DMSO was also added to each well to dissolve formazan crystals. The amount of MTT reduction to formazan in cells was measured using absorbance at 540 nm. Cytotoxicity of the compounds against the cancer cell line was measured using a microplate reader (Spectra Max plus, Molecular Devices, CA, USA) in the form of IC_50_. This assay was conducted in triplicate and nonlinear regression analysis was applied to calculate IC_50_ values.

Soft-Max Pro software was used to calculate the results of %inhibition (IC_50_), and the formula for calculating percent inhibition is shown in (Eq. 1).

1 $$\:\%inhibition=\frac{100-Mean\:of\:O.D\:of\:test\:compound-Mean\:of\:O.D\:of\:negative\:control}{Mean\:of\:O.D\:of\:positive\:control}$$

## In Silico docking studies

### Ligand preparation

The 2D molecular structures of the thiosemicarbazide and triazole analogues were generated on licensed software ChemOffice. Erlotinib was used as a standard compound and its structure was procured from the PubChem library’s database (https://pubchem.ncbi.nlm.nih.gov/). Chem 3D 16.0 was used to transform these structures into 3D forms. The Merck Molecular Force Field 94 (MMFF 94) was used for energy minimization of the target ligands, and were stored in PDB file format.

### Protein selection and preparation

For the current study, p53 (PDB ID 5G4M)^55^ and p21 proteins (PDB ID 2CDZ)^56^ with a resolution of 1.38 Å and 2.3 Å in the form of 3D crystal structure were retrieved from the RCSB Protein Data Bank (https://www.rcsb.org/). Atomic coordinates involving water molecules, heteroatoms and co-crystal ligands were removed from the PDB file by using Discovery Studio Visualizer (DSV) 2021, resulting in the separation of protein structural inhibitor SPDBV 4.2 was applied for energy minimization of protein.

### PDBQT files initialization and preparation

The generated protein molecule in PDB format was imported into the AutoDock Tool (1.5.7) workstation (http://autodock.scripps.edu). Polar H-atoms were added to the target protein to enable optimal tautomeric states and amino acid residue ionization. The Kollman and Gasteiger charges were estimated by translating the protein structure from PDB to PDBQT format. Similarly, ligand structures in PDB format were imported into the workspace, and the torsion tree was defined by root selection and number of torsions, as well as rotatable bonds, before being converted to PDBQT format. The file format was then submitted to docking studies. Previously, initial working directory was created in the selected location.

### Grid box formation

It is crucial to determine protein’s active sites in molecular docking to proceed with computations. The defined grid box dimensions of the p53 protein engaged the amino acid chain A residues PHE 109, LEU 145, TRP 146, VAL 147, THR 150, PRO 151, CYS 220, GLU 221, PRO 222, PRO 223, ASP 228, CYS 229 and THR 230. The grid dimensions were separated by 0.375 Å. The target ligands were oriented centrally in a rectangular box measured at x = 91.384, y = 95.654, z=−42.765, with the coordinates 56 × 60 × 54 xyz points. For p21 protein, the identified residues were ILE 327, GLY 328, GLU 329, VAL 335, ALA 348, VAL 379, MET 395, GLU 396, PHE 397, LEU 398, GLU 399, GLY 401, ALA 402, LEU 447, SER 457, with the grid parameters and center of 64 × 52 × 70 and x = 44.100, y = 32.502, z = 20.666 along with a spacing of 0.375Å, respectively. These variables were employed for generating configuration files. The protein binding sites were enclosed in grid box; rigid-flexible docking was executed.

### Autodock Vina

The ligand-protein binding model is evaluated using AutoDock Vina v1.5.7^57^. This method depicts the interaction model with minimized energy required by systemically evaluating the system’s degrees of freedom. Vina outperforms AutoDock4 (AD4) in docking estimation by using Broyden–Fletcher–Goldfarb–Shanno (BFGS) algorithm and empirical scoring approach. It generally refines the search space on the basis of criteria and resets the mode number, energy range, and exhaustion to their preset levels. Higher exhausted numbers increase the search duration, and also improve the precision of the results.

### Screening criteria

Docking files were assessed and visualized in PyMOL structure visualization system and DSV 2021, which displayed the position and size of the binding region, bond distance, and interactions of the standard and ligand binding model with the receptor.

### Physicochemical attributes

ChemOffice 16.0 and Swiss ADME were used to predict the physicochemical properties and drug-likeliness of synthesized compounds as molecular mass, rotatable bonds, hydrogen bond acceptors, hydrogen bond donors, hydrophobicity descriptor, and topological polar surface area^58^. The drug likeness was evaluated using the Lipinski rule of five, Verber, Ghouse filter, Muegge and Egan^59^.

### ADMET studies

Swiss ADME, pkCSM, and Pre ADMET server assess the ADMET properties of synthesized compounds. Structure-based explorations are used to determine ADMET characteristics in these resources^60^.

### Brain or intestinal estimated permeation (Boiled-Egg)

The BOILED-Egg plot evaluates a drug’s ability to permeate the BBB and GI tract. This approach was used to assess the drug-likeness of synthetic substances. A compound’s bioavailability is determined by two factors: lipophilicity (WLOGP) and TPSA. The Swiss ADME web server uses TPSA and WLOGP data to generate a BOILED-Egg plot^61^.

### DFT studies

Density Functional Theory (DFT) is theoretical quantum chemistry approach used to carried out structural calculations, elucidate reaction pathways, kinetic and energetic stability, evaluate molecular interactions, and the electronic properties of ligands. Gaussian 09 software program with B3LYP functional and SDD level of theory was employed for geometric optimization and theoretical calculations and Gauss View 6.0 was used to facilitate the visualization.

### Ethics approval and consent to participate

All methods were carried out in accordance with relevant guidelines and regulations. This study utilized an established commercial cell lines and did not involve human participants; primary tissue samples, ethical approval and informed consent were not required.

### Selected spectral data of products

#### 2-(furan-2-carbonyl)-N-(4-(trifluoromethyl) phenyl) hydrazine-1-carbothioamide 3a

White solid. Yield: 89%, Rf value: 0.56 (hexane, ethyl acetate 7:3), Isolated yield by mass: 28.5 mg; m.p. 211 °C. FT-IR νmax (cm^− 1^): 3180, 3225, 3400 (NH stretching), 2990 (ArCH stretching), 1320 (C-N), 1110 (C-F), 1090 (C = S). ^1^H-NMR (600 MHz, MeOD-d_4_): δ 6.666 (m, 1H), 7.279–7.286 (d, 1H, J = 3.6 Hz), 7.64 (d, 1H, J = 8.7 Hz), 7.77 (m, 3 H, H-1). ^13^C NMR (150 MHz, DMSO-d6): δ 113.1, 117.0, 124.8, 126.4, 126.6, 143.96, 147.08, 147.60, 160.32, 160.3, 176. 8, 184.0. EI-MS: m/z [M] calc. For C_13_H_10_F_3_N_3_O_2_S: 329.3; found 329.2, [M^+^].

#### 2-(furan-2-carbonyl)-N-(4-nitrophenyl) hydrazine-1-carbothioamide 3b

Off white solid. Yield: 71%; Rf value: 0.52 (hexane, ethyl acetate 7:3), Isolated yield by mass: 22.8 mg; m.p. 220 °C. FT-IR νmax (cm^− 1^): 3180, 3225, 3400 (NH stretching), 2990 (ArCH stretching), 1320(C-N), 1110 (C-F), 1090 (C = S). ^1^H-NMR (600 MHz, DMSO-d_6_): δ 6.7 (s, 1H, H-2), 7.27 (s, 2 H, H-3), 7.9 (m, 6 H, H-1, H-4, H-9, H-10), 8.22 (d, 2 H, J = 9.2 Hz, H-7, H-8), 10.11 (brs, 1H, H-7), 10.16 (brs, 1H, H-5), 10.54 (s, 1H, H-6). EI-MS: m/z [M] calc. For C_12_H_10_N_4_O_4_S: 306.3; found 272.2 [M^+^- H_2_S].

#### N-(3,5-bis(trifluoromethyl)phenyl)−2-(furan-2-carbonyl) hydrazine-1-carbothioamide 3c

White solid. Rf value: 0.54 (hexane, ethyl acetate 7:3), 193 °C. FT-IR νmax (cm^− 1^): 3100, 3150, 3320 (NH stretching), 2940 (ArCH stretching), 1340(C-N), 1100 (C-F), 1040 (C = S). ^1^H-NMR (600 MHz, MeOD-d_4_): δ 6.667–6.673 (d, 1H, J = 3.6 Hz), 7.292–7.298 (d, 1H, J = 3.6), 7.764–7.767 (d, J = 3.6, 2 H), 8.26 (s, 2 H). ^13^C NMR (150 MHz, MeOD-): δ 113.13, 117.18, 119.2, 122.02, 123.82, 125.63, 127.4, 132.42, 132.64, 142.2, 146.69, 147.13, 160.4, 184.28. EI-MS: m/z [M] calc. For C_14_H_9_F_6_N_3_O_2_S: 397.3; found 363.1 [M^+^- H_2_S].

#### 5-(furan-2-yl)−4-(4-(trifluoromethyl) phenyl)−2,4-dihydro-3 H-1,2,4-triazole-3-thione 4a

Off-white solid. Yield: 81%; Rf value: 0.50 Isolated yield by mass: 11 mg; m.p 201 °C. FT-IR νmax (cm^− 1^): 3180 (NH stretching), 2987 (ArCH stretching), 1270(C-N), 1120 (C-F), 1140 (C = S). ^1^H-NMR (600 MHz, DMSO-d_6_): δ 6.653–6.659 (d, J = 3.78, 1H), δ 7.278–7.286 (d, J = 3.66, 1H), 7.62–7.64 (brs, 3 H), 7.75–7.80 (m, 4 H).^13^C NMR (150 MHz, MeOD-d_4_): δ 113.1, 117.01, 124.8, 126.4, 126.6, 143.9, 147.07, 147.61, 160.3, 184.08. EI-MS: m/z [M] calc. For C_13_H_8_F_3_N_3_OS: 311.2; found 311.0, [M^+^].

#### 5-(furan-2-yl)−4-(4-nitrophenyl)−2,4-dihydro-3 H-1,2,4-triazole-3-thione 4b

White solid. Yield: 78%; Rf value: 0.51 Isolated yield by mass: 10.6 mg; m.p. 209 °C. FT-IR νmax (cm^− 1^): 3100, 3150, 3320 (NH stretching), 2940 (ArCH stretching), 1340(C-N), 1100 (C-F), 1040 (C = S). ^1^H-NMR (600 MHz, DMSO-d_6_): δ 6.63–6.655 (m, 1H), 6.72–6.73 (m, 1H), 7.19–7.20 (m, 1H), 7.72 (brs, 1H), 7.82–7.83 (m, 2 H), 7.90–7.92 (d, 2 H, J = 7.2 Hz), 8.143–8.157 (brs, 2 H, H-6), 8.29–8.31 (m, 1H). EI-MS: m/z [M] calc. For C_12_H_8_N_4_O_3_S: 288.2; found 289.1, [M + H] ^+^.

#### 4-(3,5-bis(trifluoromethyl) phenyl)−5-(furan-2-yl)−2,4-dihydro-3 H-1,2,4-triazole-3-thione 4c

Off-white solid. Yield: 79%; Rf value: 0.56 Isolated yield by mass: 10.77 mg; m.p 180 °C. FT-IR νmax (cm^− 1^): 3200 (NH stretching), 2990 (ArCH stretching), 1280(C-N), 1120 (C-F), 1150 (C = S). ^1^H-NMR (600 MHz, MeOD-d_4_): δ 6.66 (s, 1H, H-4), 7.29 (s, 1H, H-), 7.71–7.83 (m, 2 H), 8.26 (brs, 2 H). ^13^C NMR (150 MHz, MeOD-d_4_): δ 113.2 (C-2), 114.41 (C-3), 117.08 (C-10), 122.0 (C-13), 125.6 (C-14), 132.10 (C-9), 132.42 (C-11), 132.64 (C-8), 132.86 (C-12), 140.2 (C-7), 142.7 (C-1), 147.6 (C-5), 145.91 (C-4), 160.20 (C-6). EI-MS: m/z [M] calc. For C_14_H_7_F_6_N_3_OS: 379.2; found 379.1, [M^+^].

## Results and discussion

**Fig. 2:**
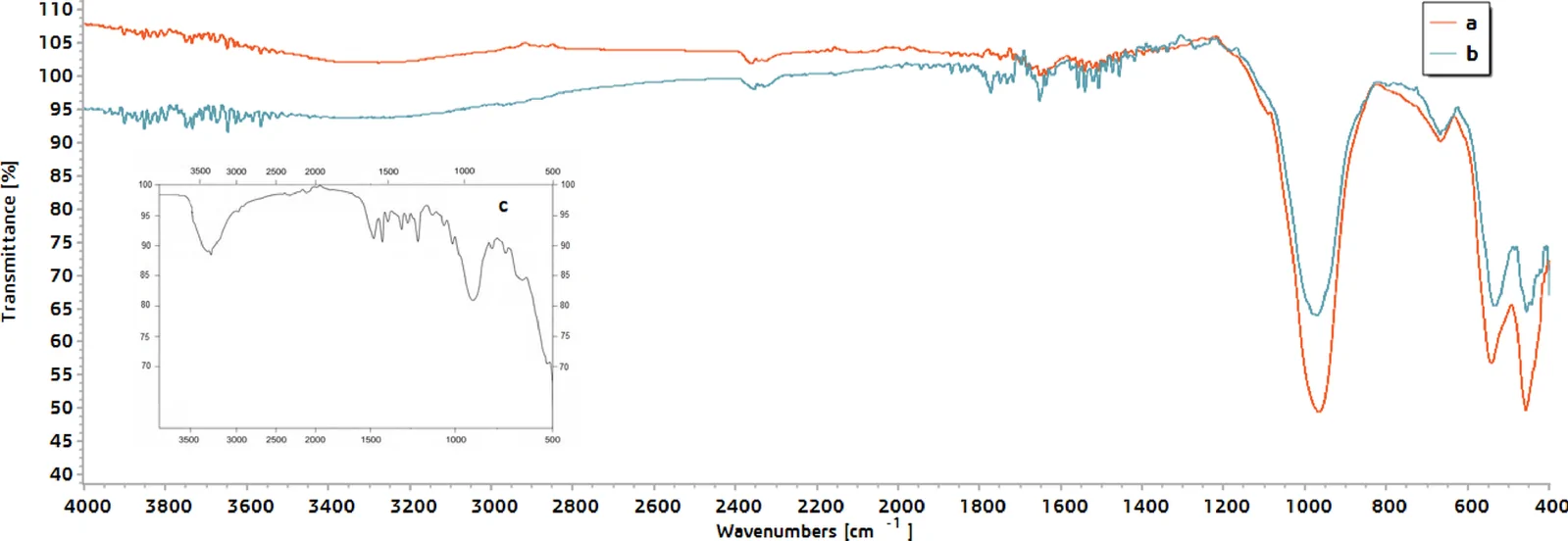
Comparative FT-IR spectra of (**a**) Na-zeolite 4 A (**b**) Co-Zeolite 4 A (**c**) Reused Co-Zeolite 4 A reflect the characteristic T–O-T (T = Si, Al) bonds remain preserved and structural stability.

For the catalyst preparation, cobalt (II) salt was impregnated on sodium zeolite 4 A framework to synthesize efficient catalyst for triazole synthesis. To gain insights into the properties of catalyst, FTIR, SEM, EDX, XRD, TGA/DSC and BET analysis was performed. The FTIR spectra of zeolite 4 A and Co-Zeolite 4 A (Fig. 2) confirm that the metal was effectively supported onto the LTA-type aluminosilicate framework. The Na zeolite-4 A exhibits a broad O–H stretching band at 3400 cm⁻¹, assigned to adsorbed and framework-coordinated water, the along with H–O–H bending band at 1653 cm⁻¹, the characteristic T–O (Si, Al) asymmetric stretching at 965 cm⁻¹, the double-ring vibration at 544 cm⁻¹, and the T–O bending mode at 456 cm⁻¹. After cobalt exchange, the framework bands shift slightly to 970, 532, and 457 cm⁻¹, respectively, indicating minor lattice distortion and stronger framework interactions due to the replacement of Na⁺ by Co²⁺ ions, while the H–O–H bending band remains unchanged, suggesting that the overall water coordination environment within the cages is largely retained. Noticeable changes occur in the hydroxyl region, where the broad O–H stretching broadens and increases in intensity, reflecting stronger interaction of water with Co²⁺ and the formation of additional Lewis-acidic sites.

The recycled Co-Zeolite 4 A was interpreted in relation to the fresh in the FT-IR spectrum, having characteristics peaks at 1611.56, 977, and 518.42 cm⁻¹ with no significant change in intensity as shown in Fig. 2 (c). The fresh and recovered catalyst spectra are in close agreement and indicate the active metal support interaction, and aluminosilicate framework remains preserved during catalysis, affirming the stability of Co-Zeolite 4 A under reaction parameters.

**Fig. 3:**
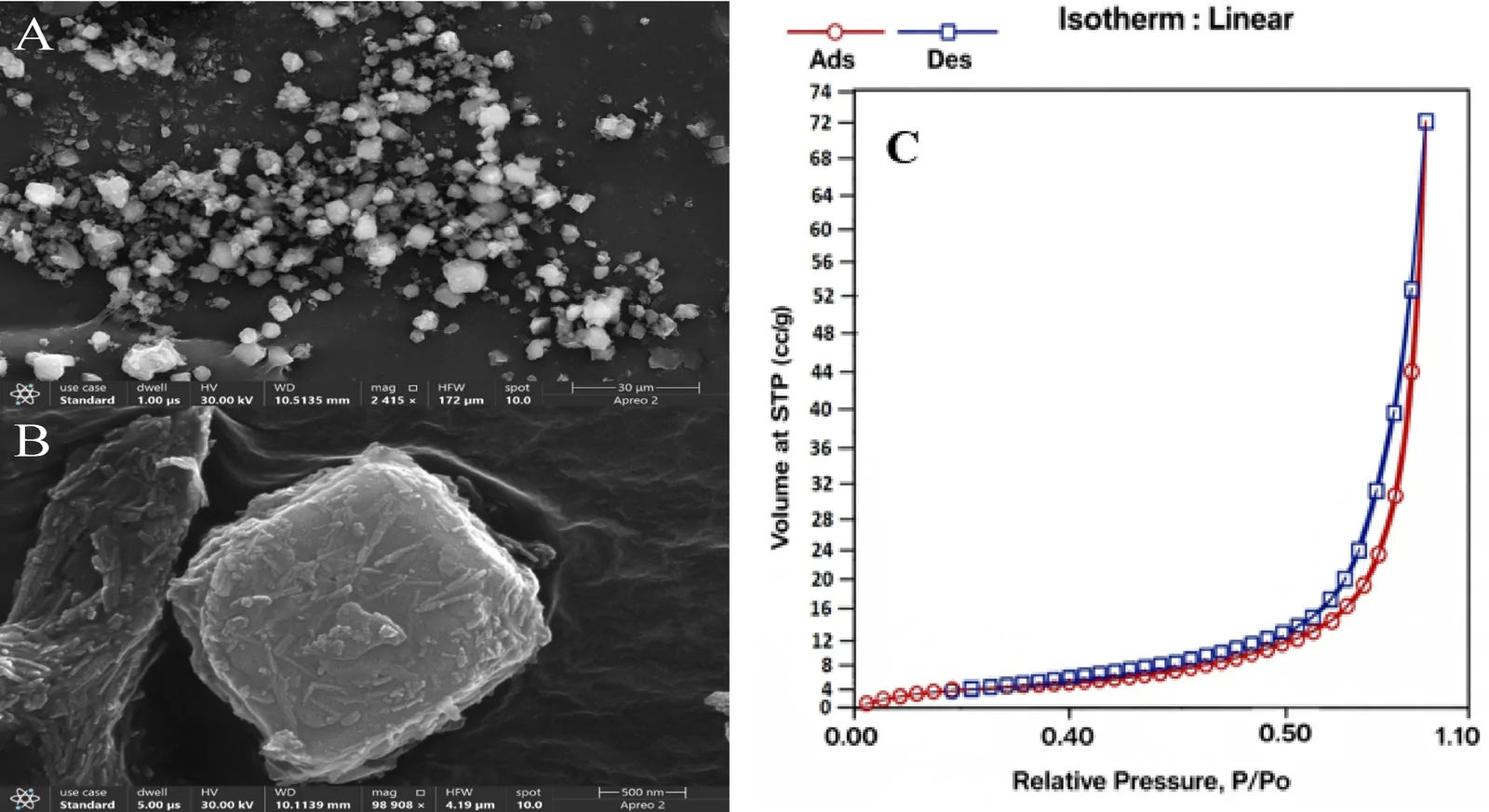
SEM images of Co-Zeolite 4 A reveal cuboctahedral morphology at (**A**) 30 µ m and (**B**) 500 nm magnifications. (**C**) N_2_ absorption and desorption isotherm illustrating reduced surface area of Co-Zeolite 4 A.

**Fig. 4:**
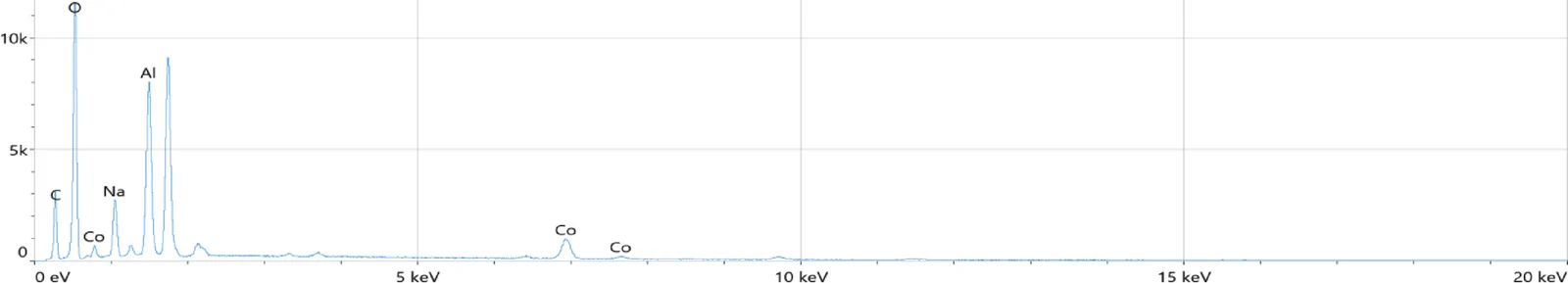
EDS diagram illustrating Co impregnation in Co-Zeolite 4 A with 3.6% cobalt content.

Surface topography, morphological features, and composition of Co-Zeolite 4 A catalyst using SEM–EDX mapping, evident the cubooctahedral-shaped zeolite crystal and uniformity in cobalt dispersion across their surface and the particle size was in the range of 500 nm to 1 μm and, and the cobalt content was 3.6% as shown in Figs. 3 and 4. The crystal intergrowth on the morphological surface of the catalyst are commonly reported in zeolite 4 A and is attributable to their synthetic conditions^62^. This supports the idea that well-dispersed cobalt particles may facilitate the accessibility of active sites as it is crucial for enhanced catalytic activity. SEM morphology and FT-IR spectrum of reusable catalyst confirmed no significant change, reflecting better structural stability and resistance to degradation.

Diffraction peaks of 4 A Na-zeolite were observed at 2θ (degree) of 7.3, 10.3, 12.6, 16.2, 20.5, 21.8, 24.1, 26.2, 27.2, 30.08, 34.3, 41.6, 44.3 and 52.7, which corresponded to the crystalline planes (200), (220), (222), (421), (440), (610), (622), (640), (642), (644), (850), (881), (1211) and (1421) respectively (according to JCPDS 39–0222)^63,64^. The XRD pattern of Co-Zeolite 4 A showed most of the above-mentioned peaks with noticeable increased in intensity, as well as no distinct Co–related diffraction peaks were observed, confirming that cobalt ions have become part of the zeolite framework through ion exchange, rather than forming separate cobalt compounds as shown in Fig. 6. Sharper peaks in the XRD pattern confirmed that the Co-Zeolite 4 A catalyst retains its crystallinity after metal loading. Scherrer’s formula was used to determine the average crystallite size as, K = 0.9λ/β·cos θ equivalent to 56.2 nm (Supplementary Table S-1) for Co-Zeolite 4 A, where K represents crystallite size, λ is the 1.5406 nm, β is the peak’s full width at half maximum (FWHM) and, θ is the diffraction angle^65^. The large particle size observed in SEM observations elaborate that the XRD provides size of coherently scattering domains of Co-Zeolite 4 A particles^66^. Based on 4 A Na-zeolite with space group of Fm-3c the cell parameter of Co-Zeolite 4 A was expressed by Eq. 2:

2 $$\:\frac{1}{{d}^{2}}=\frac{({{h}^{2}+k}^{2}+{l}^{2})}{{a}^{2}}$$

Where h, k, and l are the Miller indices of the diffraction planes, and d is the spacing distance, a is cell parameter. The computed cell parameters altered insignificantly reflecting that the introduction of metal negligibly effect interplanar spacing of Co-Zeolite 4 A. Decrease in FWHM values of the main diffraction peaks signifies larger crystallite size of Co-Zeolite 4 A, concluding that the addition of cobalt strengthened the crystalline framework of 4 A molecular sieves, which compensates for the minor reduction in support surface area^67^. It is expected that the availability of active sites with high metal dispersion after Co impregnation was unaffected by an increase in crystallite size, as demonstrated by the significant catalytic activity of Co-Zeolite 4 A in the model process.

Decrease in surface area from 10.1 m^2^/g to 9.4 m^2^/g after cobalt impregnation, confirming metal anchoring on to the framework in BET analysis (Supplementary Figure S-1 and S-2). The impregnated Co cation in the structure cause the pore blocking effect that results in smaller surface area. The N_2_ adsorption–desorption isotherm is as shown in Fig. 3. The apparent hysteresis loop could be arising from interparticulate porosity in microporous zeolite 4 A materials is attributed to the capillary condensation in inter-crystalline spaces and diffusion limitations of nitrogen rather than intrinsic mesoprosity^68,69^. XRD patterns indicated that the crystalline framework of the 4 A molecular sieves remained intact after cobalt incorporation.

Co-Zeolite 4 A displayed the slight weight loss (15%) that continues up to around 120 °C associated with the removal of physically adsorbed water and hydroxyl groups on the zeolite surface followed by the major weight loss (22%) up to the temperature of 267 °C, attributed to the strongly bounded water in zeolite framework and dehydration of hydrated Co^2+^ species as illustrated in TGA/DSC thermogram. After the complete dehydroxylation processes at the temperature of 358 °C, a gradual weight gain (3%) was evident above this temperature, associated with the oxidation of supported cobalt (Co^2+^ to Co^3+^) due to oxygen uptake. The initial endothermic events observed in the DSC curve are consistent with the mass loss stages followed by the exothermic phase at high temperatures, demonstrating Co-Zeolite 4 A ‘s thermal resilience and structural integrity (Fig. 7). The results support the efficiency and reusability of the catalyst under applied reaction conditions.

**Fig. 5:**
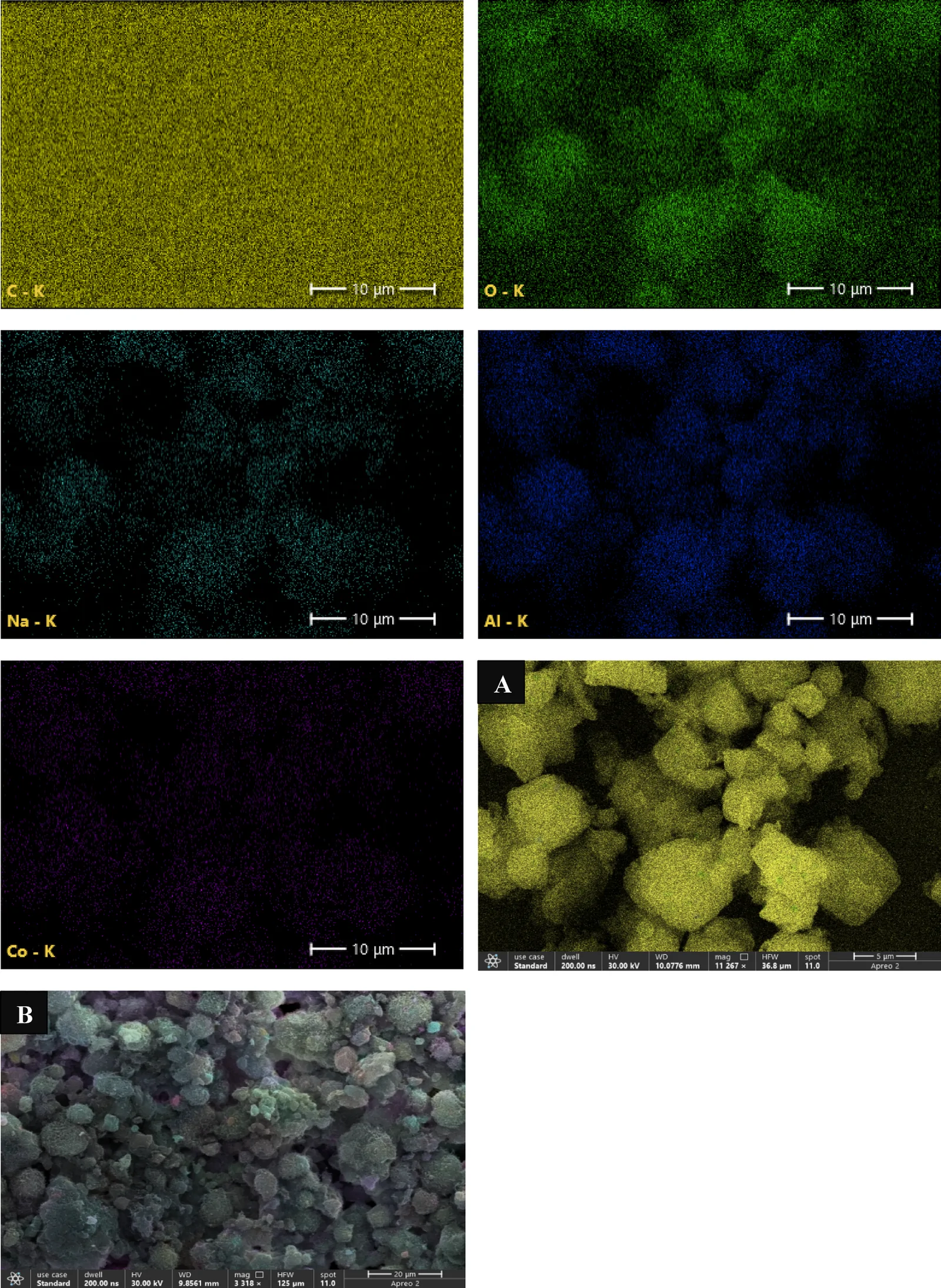
Element mapping of investigated site, SEM image of (**A**) Co-Zeolite Catalyst (**B**) Reused catalyst.

**Fig. 6:**
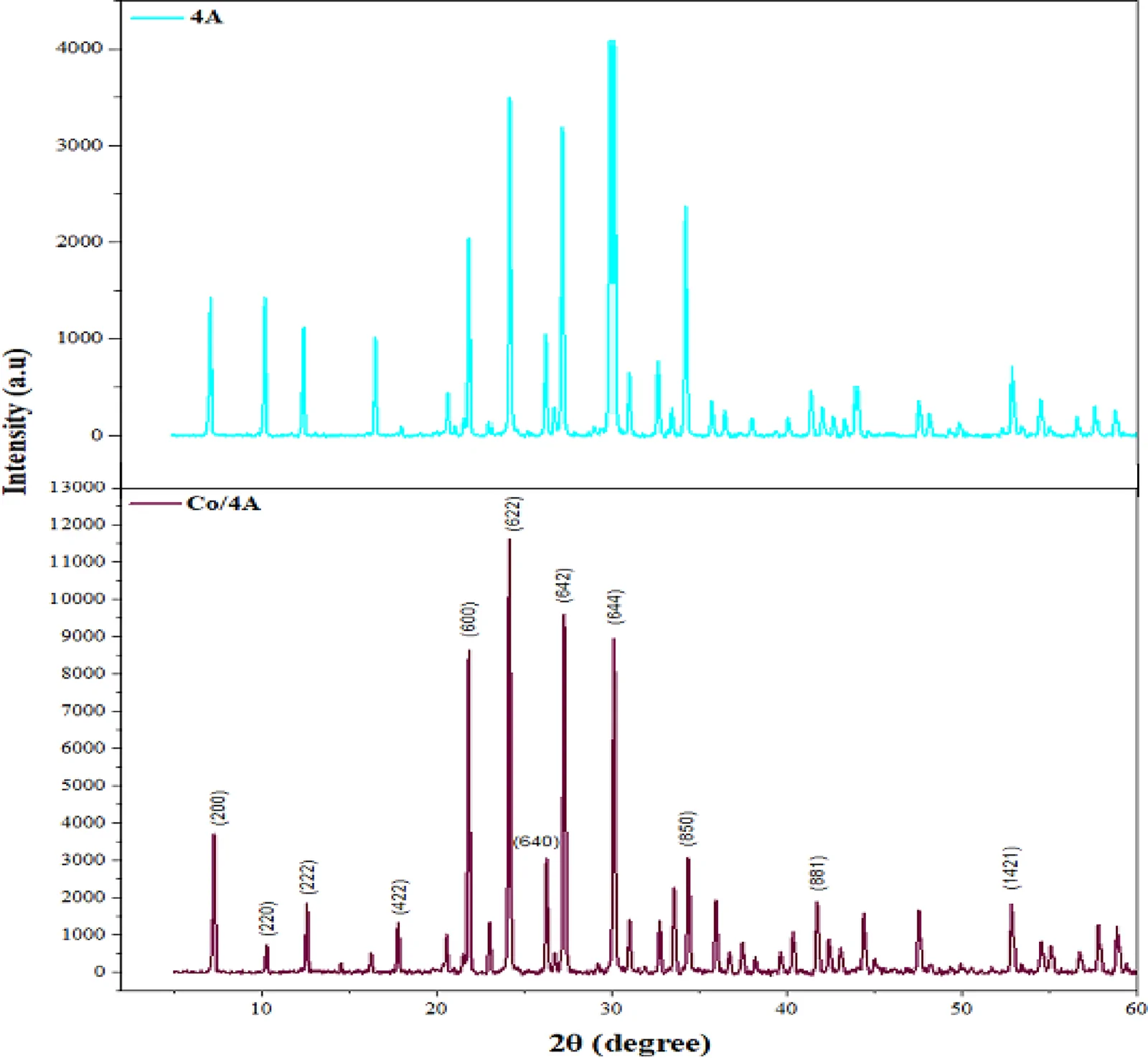
X-ray diffractograms of Zeolite 4 A (blue) and Co/Zeolite 4 A (indigo) showed successful metal incorporation, maintained the zeolite framework.

**Fig. 7:**
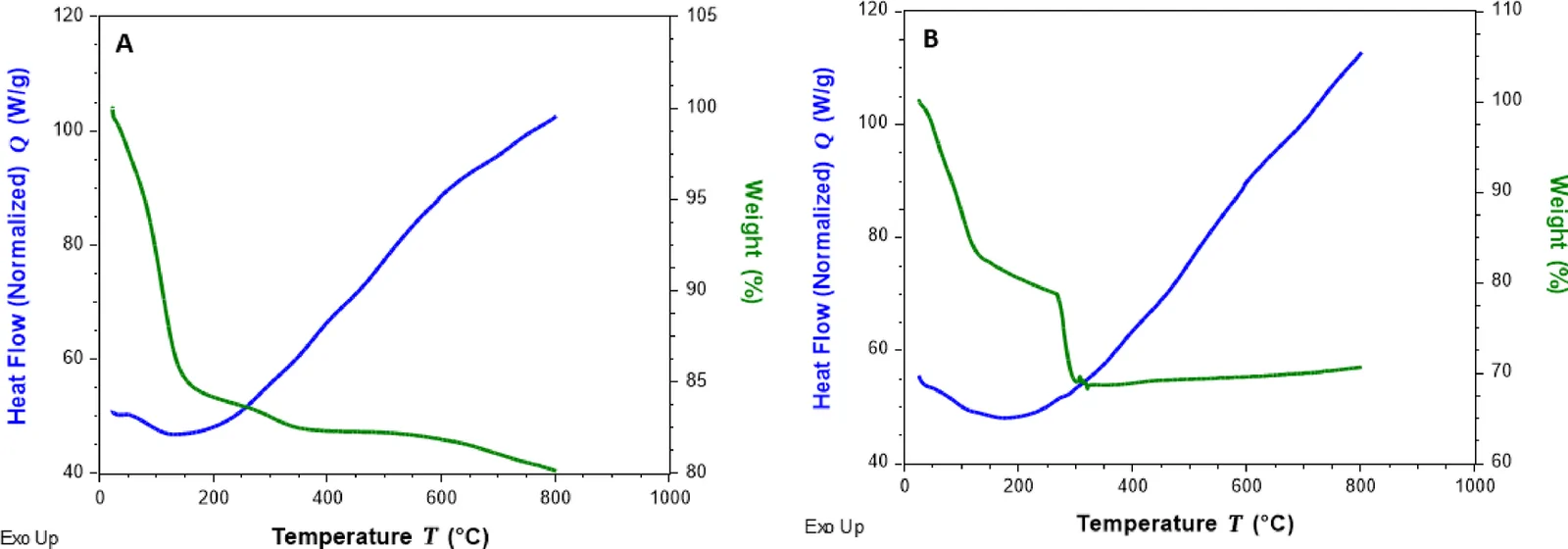
TGA-DSC spectra reveal more gradual weight loss of **B)** Co-Zeolite 4 A catalyst as compared **A)** Na-zeolite 4 A.

The 2-furyl-1,2,4 triazole-3-thione derivatives were synthesized following a modified reported protocol as illustrated in Fig. 7. In this reaction, 2-furoic hydrazide (1) and substituted phenyl isothiocyanates (2a-c) undergo nucleophilic addition-condensation reaction in which terminal –NH of hydrazide attacked electrophilic carbon of isothiocyanate and produce acyl thiosemicarbazides (3a-c) as an intermediate. In the subsequent step, treatment of sodium hydroxide induces intramolecular cyclization of the intermediate followed by tautomerization, yielding derivatives of 1,2,4 triazoles.

Catalytic activity of Co-Zeolite 4 A was assessed on the model reaction between 2-furoic hydrazide (1) with 4-trifluoromethyl phenyl isothiocyanate (2a) and to find the optimized reaction conditions, various parameters consisting of solvent, catalyst, amount of catalyst, and temperature (Fig. 7). According to the results summarized in Table 1, the significant outcomes was obtained from Co-Zeolite 4 A as a catalyst in aqueous ethanol solvent condition and yielding 89% 3a under the reflux conditions for 1 h at 40 ℃, low yield was obtained in catalyst free conditions under refluxing in methanol for 24 h. The intermediate polarity of ethanol and water promote hydrogen bonding and optimized polarity relative to methanol system. The effect of solvent in the catalytic reaction facilitate the reactants dispersion, mass transfer process and alters the reaction kinetics^70^. Subsequently, 10 mg Co-Zeolite 4 A catalyst in ethanol/water 1:1 catalyzed the reaction with high product yield and RT. It is expected that the lower yield with 3 mg Co-Zeolite 4 A was owing to their less catalytic sites, and significant efficacy for 10 mg catalyst could be ascribed to the well-dispersed and surface-exposed cobalt active sites on the zeolite.

**Fig. 8:**
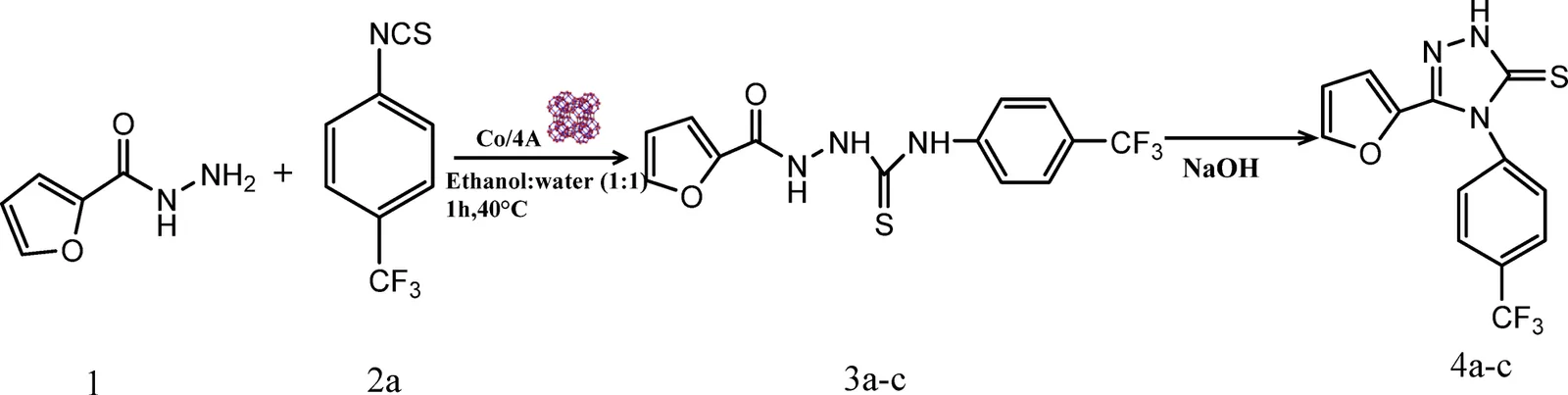
Co-Zeolite 4 A catalyzed synthesis of 5-(furan-2-yl)−4-(4-(trifluoromethyl) phenyl)−2,4-dihydro-3 H-1,2,4-triazole-3-thione.

To demonstrate the prominence of Co-Zeolite 4 A, we have processed the same reaction with Ni-Zeolite 4 A and Fe-Zeolite 4 A (Table 1). Among the catalysts tested, the Co-Zeolite 4 A catalyst system exhibited superior activity, affording the corresponding products in high yields under similar reaction conditions whereas replacing cobalt with iron and nickel led to considerably lower conversions owing to cobalt balanced Lewis acidity and strong dispersibility on zeolite support. The same reaction was performed in support zeolite 4 A as catalyst, further verifying the role of cobalt in the catalytic cycle. Mechanistically, the reaction likely proceeds via coordination activation of the carbonyl and amine substrates on Co(II) sites, followed by intramolecular cyclization and dehydration to furnish the heterocyclic framework.

Following the reaction parameters optimization to describe this procedure, the current experimental method was analyzed with diverse phenyl isothiocyanates (2a-c) in the presence of Co-Zeolite 4 A under consistent parameters. As we have anticipated, the optimized strategy worked well in the reaction of different phenyl isothiocyanates containing various electron-withdrawing groups at different positions, such as CF_3_ and, NO_2_ (Fig. 8). Subsequent treatment with sodium hydroxide induced intramolecular cyclization, where deprotonation of the hydrazinic nitrogen facilitated nucleophilic attack on the thiocarbonyl carbon, to yield different 1,2,4 triazoles derivatives. This work may result in intriguing chemistry using innovative methods for synthesis of promising pharmacophores. A significant aspect of this reaction relative to reported methods is the use of green solvent and shorter reaction time.

**Fig. 9:**
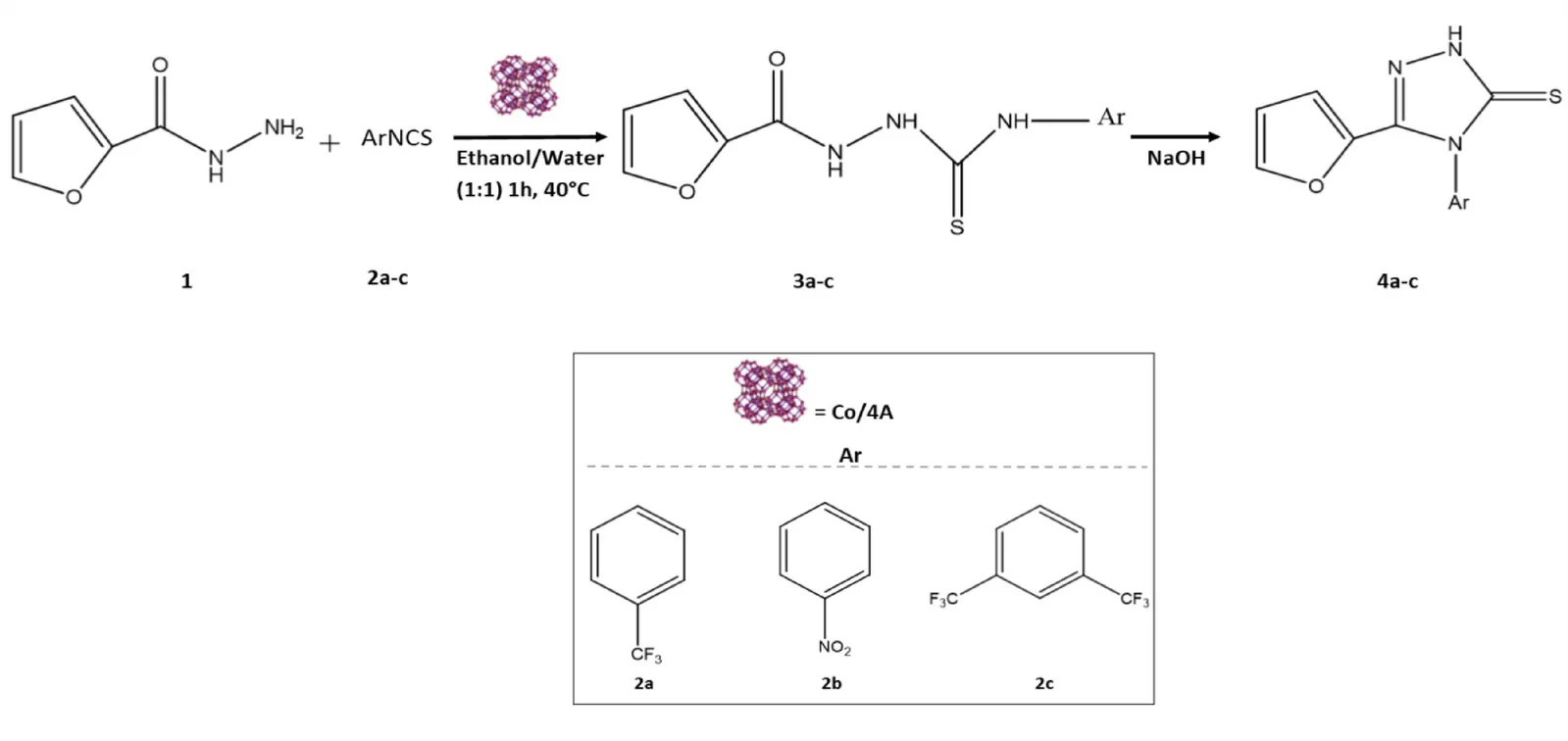
Co-Zeolite 4 A catalyzed synthesis of Co-Zeolite 4 A catalyzed thiosemicarbazide (3a-c) and 1,2,4 triaole-3-thione scaffolds (4a-c).

**Table 1:** Optimization of reaction parameters^a^.

Entry	Catalyst	Solvent	Temperature (˚C)	Reaction time	Yield (%)^b^
1	No catalyst	Methanol	60	24 h	47
2	No catalyst	Ethanol	40	20 h	52
3	No catalyst	Ethanol: water (1:1)	40	20 h	68
4^c^	Co-Zeolite 4 A	Ethanol: water (1:1)	40	1 h	80
5^d^	Co-Zeolite 4 A	Ethanol: water (1:1)	40	1.5 h	79
6^e^	Co/Zeolite 4 A	Ethanol: water (1:1)	40	1 h	89
7	Ni- Zeolite 4 A	Ethanol: water (1:1)	40	8 h	71
8	Fe- Zeolite 4 A	Ethanol: water (1:1)	40	13 h	70
9	Zeolite 4 A	Ethanol: water (1:1)	40	16 h	74

^a^Reactions were carried out among 2-furoic hydrazide (0.1 mmole) and 4-trifluromethyl phenyl isothiocyanate (0.1 mmole) in aqueous ethanol system and 5 mg of catalyst.

^b^Isolated product yield. ^c^5 mg of Co-Zeolite 4A. ^d^3 mg of Co-Zeolite 4A. ^e^10 mg of Co-Zeolite 4A was refluxed.

**Fig. 10:**
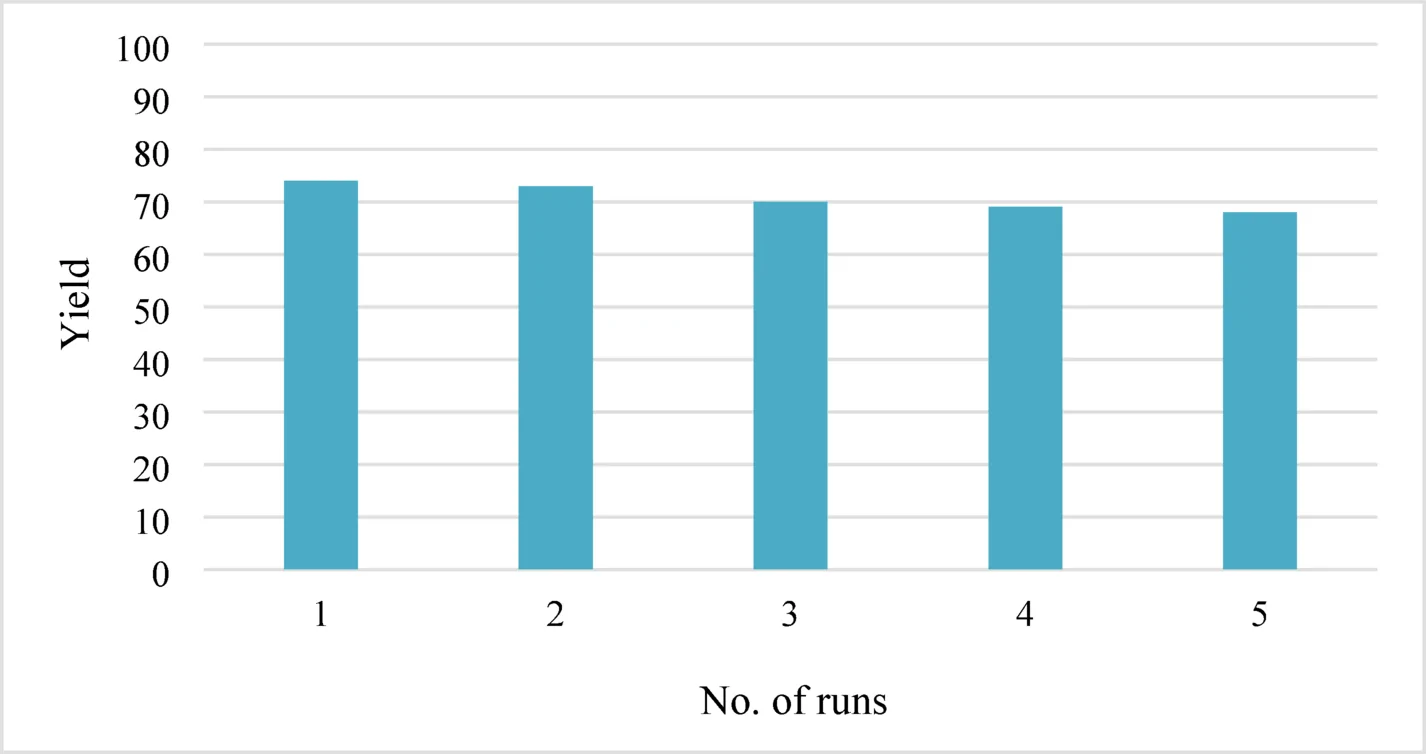
Recyclability of the Co-Zeolite 4 A catalyst for the synthesis of 3a.

**Fig. 11:**
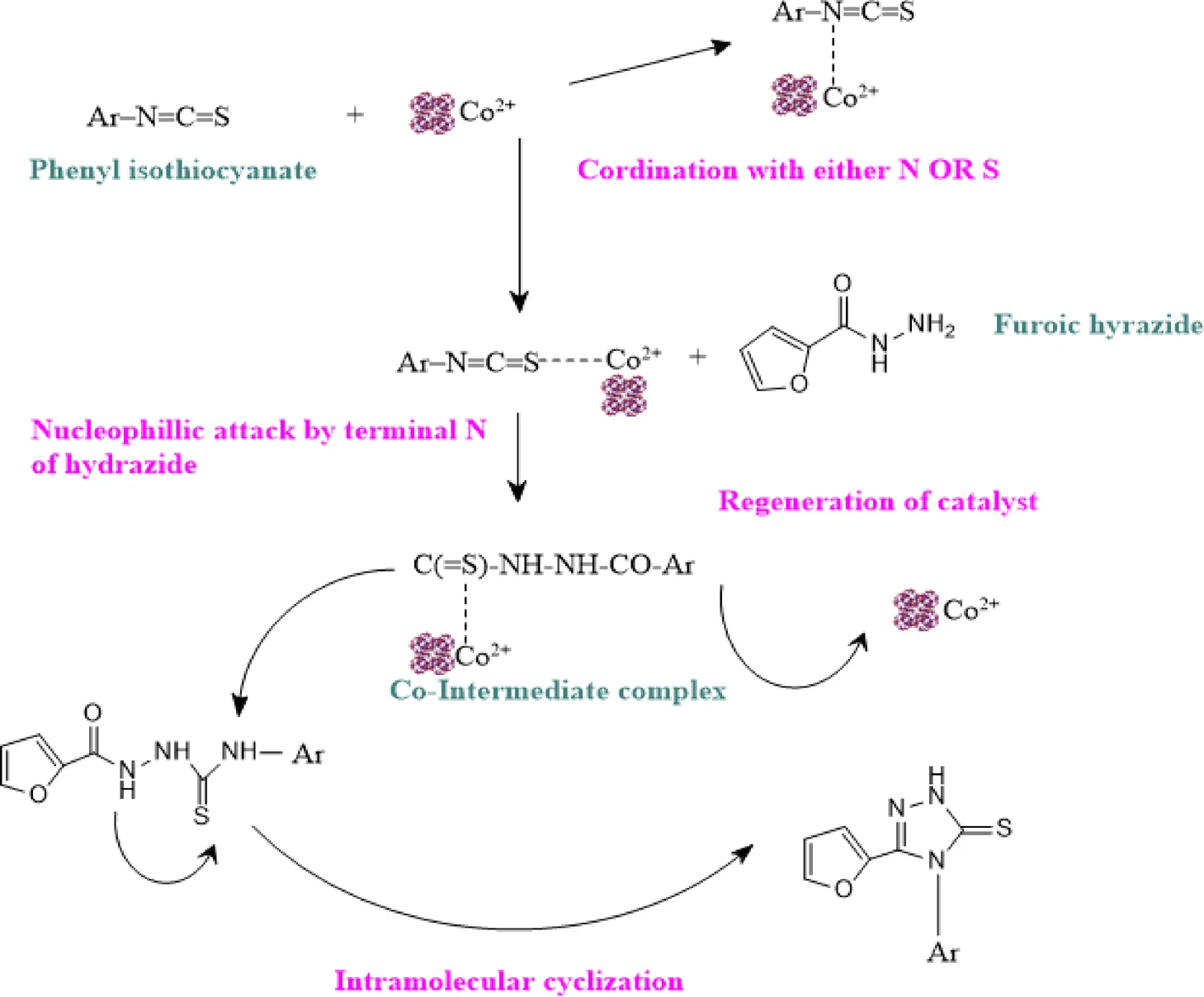
The possible mechanism for the synthesis of 3a in the presence of Co-Zeolite 4 A.

### The possible mechanism for the synthesis of 1,2,4-triazoles in the presence of Co-Zeolite 4 A

The reaction executed by activating phenyl isothiocyanate in the presence of Co-Zeolite 4 A through coordination with nitrogen or sulfur as lewis acid catalyst, where Co^2+^ active sites on molecular sieves increase the central carbon electrophilicity as shown in Fig. 11. The DFT analysis supported the idea of the possible interaction of sulfur due to high electron density as compared to nitrogen. Subsequently the nucleophilic attack by amino group of hydrazide resulting thiourea intermediate formation. The presence of zeolite lewis acid site and surface hydroxyls groups stabilized the intermediate and promotes bond rearrangement to form C(= S)–NH–NH–CO–R linkage^71,72^. The generated thiosemicarbazide detaches the catalyst and result in the regeneration of Co-Zeolite 4 A with stable framework of molecular sieves as confirmed by FTIR data of reusable catalyst.

**Fig. 12:**
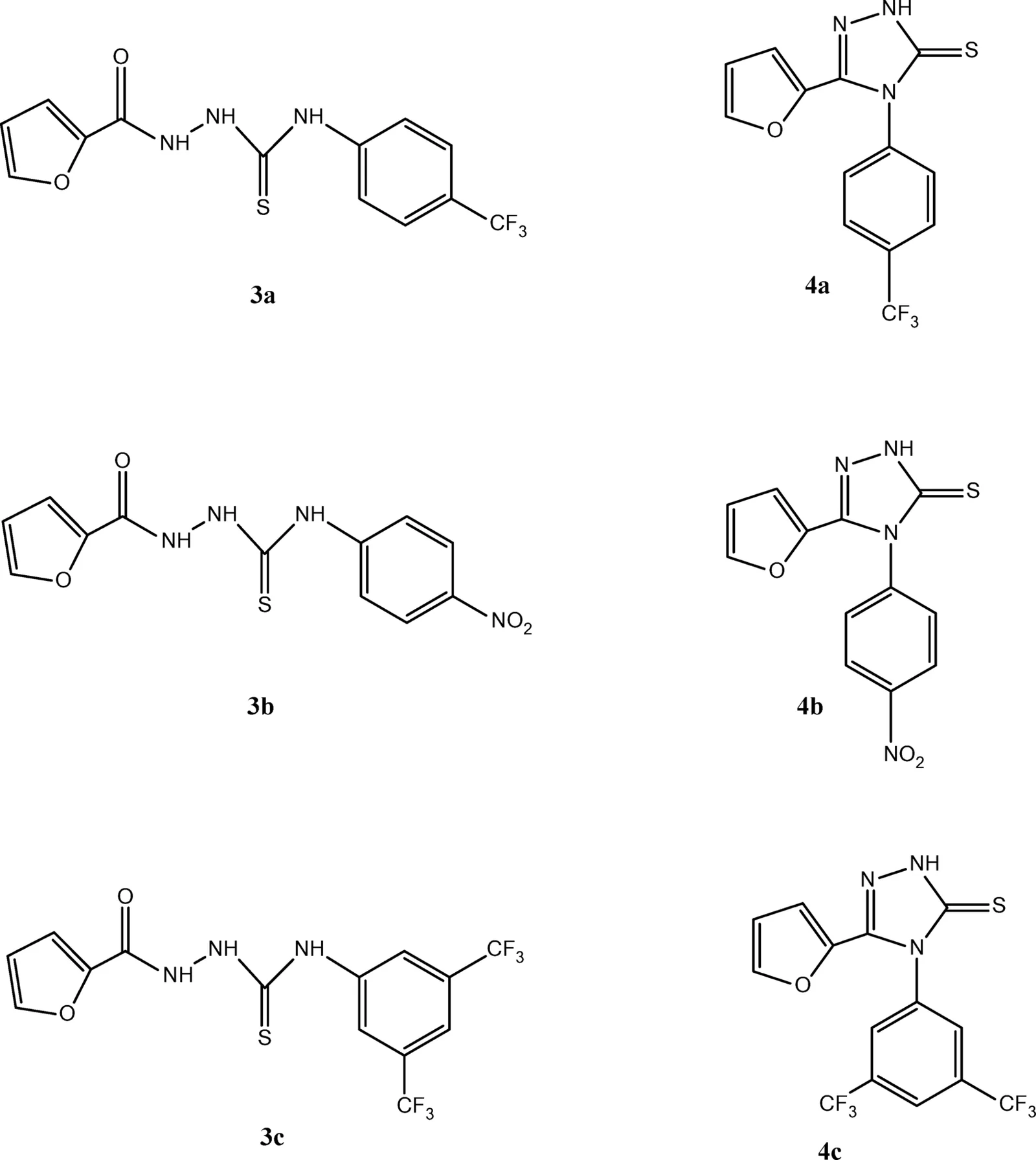
Co-Zeolite 4 A catalyzed synthesis of 2-furyl-thiosemicarbazides (3a-c) and 2-furyl-1,2,4 triazole derivatives (4a-c).

The reusability of the catalyst was assessed under the standard reaction parameters for the synthesis of 3a compound as a model reaction. The catalyst was isolated from the reaction solution, filtered and washed with ethanol thrice to remove reactants and products and dried in an oven at 120 °C for 1 h. After complete drying, the dried recovered catalyst was tested in the reactions with similar parameters. The reusability was tested in five consecutive runs, and it was inferred that Co-Zeolite 4 A can be reused in the first run no significant loss in catalytic performance. In the subsequent runs, there was a decrease in yield from 74% to 69%. The results of reusability are displayed in Fig. 10.

A comprehensive structural analysis of all synthesized products was performed using FT-IR, ^1^H NMR, ^13^C NMR, and EI-MS. FT-IR analysis of thiosemicarbazide derivatives 3a-c showed ν(N-H) stretching bands for PhNH, CSNH, and CONH at 3350–3300 cm^− 1^, 3250–3200 cm^− 1^ and 3150–3110 cm^− 1^, respectively. Furthermore, characteristic stretching bands of ν(C = S) and ν(C = O) were identified at 1650 cm^− 1^ and 1150–1010 cm^− 1^, respectively. The stretching band of ν(N-H) for CSNH of 1,2,4 triazole derivatives 4a-c was recorded at 3150–3100 cm^− 1^. The stretching band of ν(N-H) for CONH in thiosemicarbazide derivatives 3a-c diminished, whereas a weak stretching band of PhNH at 3270–3250 cm^− 1^ appeared in triazoles 4a-c.

^1^H NMR spectra of all thiosemicarbazide molecules (3a-c) have shown the signal of three protons in furan ring were found at 6.5 − 8.2 ppm and the aromatic protons at 7.2 − 8.0 ppm. In methanol the disappearance of exchangeable − NH amide protons (− CONHN = CS−NH) attributed to hydrogen/deuterium exchange, whereas these protons were recorded at 10.11 − 10.16 ppm in DMSO for compound 3b. In all triazole molecules (4a-c) emergence of downfielded signals of protons in furan and aromatic rings due to cyclization. The formation of the cyclized 1,2,4 triazole was confirmed with ^13^C NMR studies. ^13^C NMR spectra has shown that the signals belonging to C = S and C = O groups at 160.4 ppm and 180.04 to 180.28 ppm, aromatic carbons at δ122.0 and 143.9 ppm and furan ring (C1-C4) at δ 142.1–147.07 ppm, 113.1–113.2 ppm, 114.41–117.0 ppm, and 145.97–147.67 ppm, respectively The C-5 of azomethine (-C = N-) and the C-6 of thioxo (-C = S-) of 4a-c resonated at δ 160.2–160.3 ppm and 176–183 ppm. The m/z observed peaks corresponding to molecular ion peak [M] ^+^ confirm the products. The [M + H] peak was observed in mass spectrum of 4b, while [M - H_2_S] ^+^ peaks on mass spectra of 3b and 3c, typically the result of H_2_S cleavage showing the intense molecular fragment peak.

A crucial metric of cytotoxic efficacy in drug screening, the IC_50_ (half-maximal inhibitory concentration) is the concentration of a molecule needed to inhibit 50% of cell viability. By evaluating cell metabolic activity across a concentration gradient, the MTT test yields IC_50_ values that uncovers dose-dependent lethal effects. Higher potency is indicated by lower IC_50_ values. Standard MTT assay was employed to evaluate in vitro antiproliferative activity of synthesized derivatives (3a-c,4a-c) and erlotinib as standard against HeLa and PC3 cell lines. The evaluation of their cell viability assay revealed that among thiosemicarbazide and triazole derivatives, 3a, 3c, 4a, and 4c displayed potent antiproliferative activity against HeLa cell lines against while 3c was most active among all for PC3. Based on preliminary bioassay outcomes, the IC_50_ values were determined to assess potential anticancer activities. IC_50_ values (µM) as the metric were used for the evaluation of the cytotoxic potential of synthesized derivatives against cervical and prostate cancer cell lines, comparable to erlotinib, as presented in Table 2. The IC_50_ values of six different compounds were evaluated. For HeLa cell line, the two compounds with the lowest IC_50_ values were 4c (8.74 ± 0.13 µM), and 4a (22.1 ± 0.2 µM) as compared to standard anticancer drug erlotinib (41.80 µM). In PC3 cells, 3c (21.3 ± 0.93 µM), 4a (29.51 ± 1.57µM), 4c (29.51 ± 5.10 µM) found to be more active among all synthesized derivatives, whereas erolitinib displayed IC_50_ value of 15.45 µM. 1,2,4 triazole derivative 4c depicted the most potent anticancer activity, and thiosemicarbazide derivative 3b showed reduced efficiency in the screened HeLa cell lines. The compound 4a exhibited superior efficacy based on potency than standard anticancer drug erlotinib for HeLa and PC3, indicating the importance of the nitrogen-rich heteroatomic pharmacophore in synthesized derivatives.

**Table 2:** Cell viability of compounds 3a−3c and 4a−4c against HeLa and PC3 cancer cell lines by MTT assay.

IC_50_ values in µM^a^		
Compound	HeLa	PC3
3a	20.37 ± 0.13	30.24 ± 0.04
3b	NA^b^	43.6 ± 3.4
3c	35.14 ± 0.04	21.33 ± 0.931
4a	22.1 ± 0.20	29.51 ± 1.57
4b	83.18 ± 0.29	NA
4c	8.74 ± 0.13	29.51 ± 5.10
erlotinib^*c*^	41.80	15.45

^a^IC_50_: The compound’s concentration that inhibits 50% of cell progression after 48 h of drug treatment, measured by the MTT assay. The average values ± standard deviation are provided for each test, which was conducted at least three times

^b^NA: The compound exhibits IC_50_ value greater than 50 *μ*M/mL. ^c^Erlotinib as a standard drug. All expermients were performed in triplicate.

The insights of structure−activity relationship (SAR) analysis of the screened compounds indicated that the noticeable patterns were based on substituted groups positioned on the central scaffold. Among these, 4c and 4a, having a trifluromethyl group at position of 4c, exhibit the remarkable activity in both cell lines, attributed to the presence of a halogen group notably increases activity, and 4c displayed most pronounced activity, particularly in HeLa cell line. Compounds 3b and 4b with nitro groups showed lowest activity in HeLa and PC3 cell lines. These results be crucial for enhanced cellular permeability due to the lipophilic characteristics. Collectively, the highly effective compounds (4c and 4a) leverage the trifluromethyl substituents at strategic positions, elucidating their essential function in increasing antiproliferative response.

### In silico molecular docking studies

**Fig. 13:**
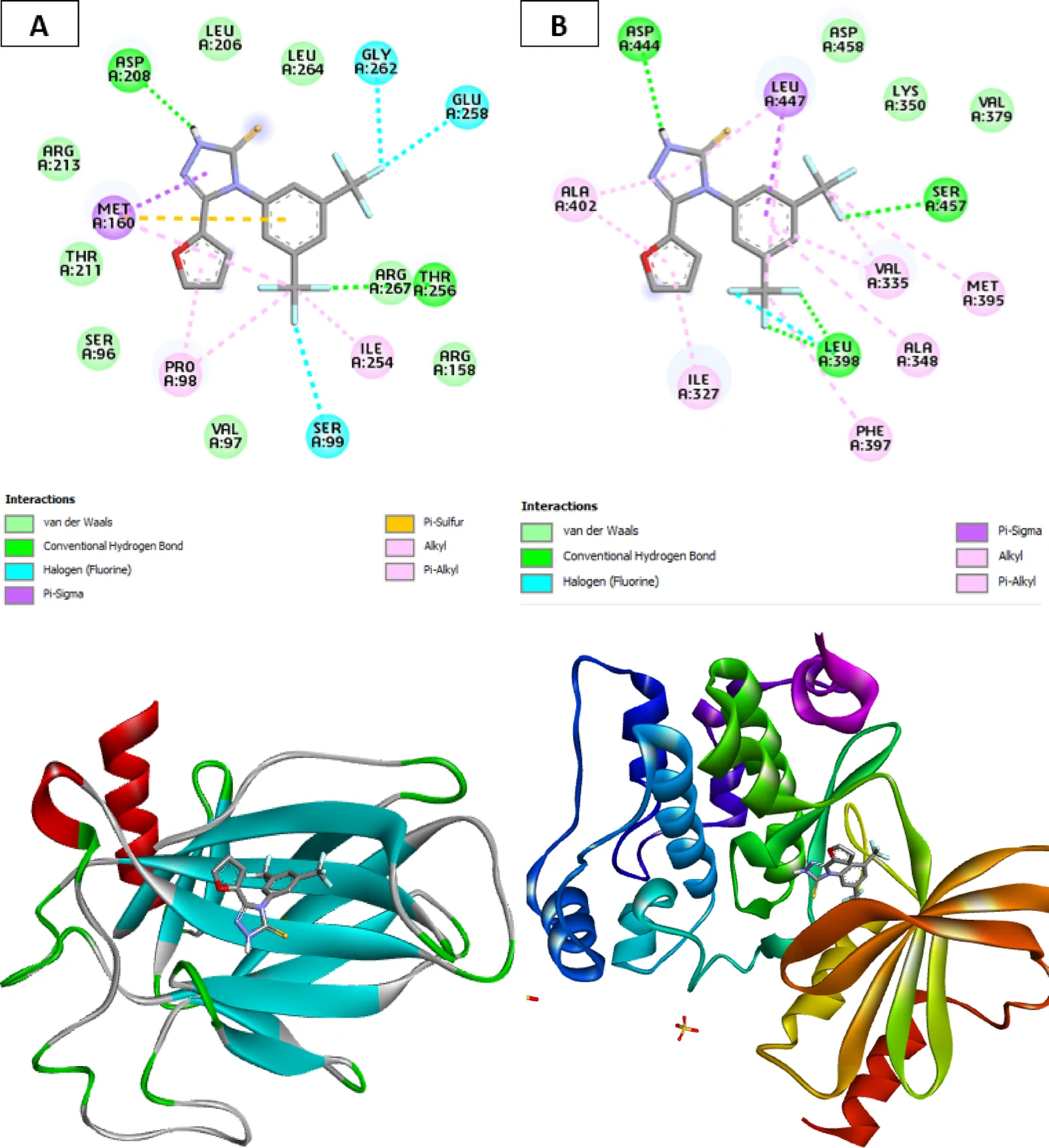
Visualization of 2D and 3D of the most active docked ligand of compound 4c using DSV 2021. (**A**) Binding models of 4c in the active sites of p53 (PDB ID 5G4M), (**B**) Binding models of 4c in the active sites of p21 (PDB ID 2CDZ).

Computational docking analysis predicted the affinity and binding pose of proposed chemical structure of compounds with the target, facilitating the drug design process. Tumor suppressor p21 and p53 proteins were chosen because of their well-established function in regulating cell growth and survival in cancer. Binding affinities of screened ligands and type of associations of the compound 4c with the target proteins are presented in Tables 3 and 4. The in silico docking studies for p21 and p53 proteins showed varying binding interaction and affinities features among screened compounds, with most surpassing the standard anticancer drug erlotinib. Figure 13 demonstrates the best ligand orientation and binding affinity at an active site anticipated from the protein-ligand model.

For p21 protein, erlotinib revealed the docking score of −7.7 kcal/mol, forming π-sigma bonds with VAL 335, LEU 447 and ILE 327, π–alkyl bonds with PHE 397, ALA 348 and LEU 398 residues. Additionally, erlotinib exhibited van der waals forces with GLU 396, MET 395, LYS 350, VAL 397, PHE 459 GLU 399, ALA 402 and GLY 401 and hydrogen bonds with ASP 458 and SER 459, and carbon-hydrogen bonds with GLU 366 and ASP 444. Among all screened ligands, 3c, and 4c displayed excellent binding affinities compared to erlotinib, with docking scores of −8.3 kcal/mol and − 9.3 kcal/mol. The binding interaction of 4c with p21 reveals the formation of π-sigma bond between phenyl moiety of compound with LEU 447, π–bonds with ALA 402, ILE 327, PHE 397, ALA 348, MET 395 and VAL 335. The compound also displayed van der Waals forces with ASP 458, LYS 350 and VAL 379, hydrogen bonds with ASP 444, SER 457 and LEU 398 and halogen (fluorine) bond with LEU 398 residues, highlighting its potential effectiveness as p21 inhibitor.

For p53, the erlotinib exhibited docking score of −6.4 kcal/mol. The compound core showed key interactions with CYS 220, GLU 221, PRO 151, PRO 222, PRO 223, van der Waals forces with ASP 228, SER 149, and THR 150 residues. The result of docking for compound 4c with p53 exhibits a docking score of − 7.7 kcal/mol attributable to one π-interactions of furan and phenyl ring with MET 160 residue. Similarly, key residues involving MET 160, PRO 98 and ILE 254 interacted through π-interactions, van der Waals forces with ARG 267, ARG 158, THR 211, SER 96, VAL 97, LEU 206, LEU 264, two conventional hydrogen bonds with ASP 208 and THR 256, and significant halogen (fluorine) interactions with GLU 258, GLY 262 through electrostatic stabilization.

The docking results of the furan-substituted thiosemicarbazides and 1,2,4 triazole derivatives with p21 and p53 target active sites reveal excellent binding affinities, specifically 4c, that possess a para-trifluoromethyl group attached to the triazole ring. The notable activity of synthesized derivatives may have attributed to the heterocyclic and hydrogen bond pharmacophoric similarities of erlotinib. A negative docking score indicates a good binding affinity of the compound to the protein’s active site. Within the binding pocket, 4c is involved in multiple intermolecular interactions, such as hydrogen bonds, hydrophobic, van der Waals, and π interactions. These ligand-protein bond formations are essential for its strong binding to the target and pharmacological effects.

**Table 3:** Comparative docking analysis of erlotinib and synthesized compounds with p21, and p53.

Entry	Compounds	Binding energy (Kcal/mol)	
		p21	p53
1	Erlotinib	−7.7	−6.4
2	3a	−7.6	−6.6
3	3b	−7.0	−6.7
4	3c	−8.3	−7.1
5	4a	−7.5	−6.8
6	4b	−6.9	−6.6
7	4c	−9.3	−7.7

**Table 4:** Binding energy and type of associations between ligand (**4c**) and the target proteins computed by molecular docking.

Target protein	Binding energy (kcal/mol)	Hydrogen bonding (Å)	Hydrophobic interactions	π-Interactions (Å)	Van der Waals
p21	−7.7	ASP 444 (3.9), SER 457 (5.12), LEU 398 (4.8)	ASP 444 (3.97), SER 457 (5.12), LEU 398 (4.81)	MET 395 (5.63), ALA 402 (5.66), ILE 327 (5.35), PHE 397 (4.81), ALA 348 (7.74), VAL 335 (4.91)	ASP 458, LYS 350, VAL 379
p53	−9.3	ASP 208 (3.87), THR 256	GLU 258 (5.0), GLY 262 (5.31), SER 99 (4.94).	MET 160 (6.48), PRO 98 (4.38), ILE 254 (4.36)	ARG 267, ARG 158, ARG 213, THR 211, SER 96, VAL 97, LEU 206, LEU 264

**Table 5:** Drug likeness rules.

Rules	Compounds					
	3a	3b	3c	4a	4b	4c
Lipinski	Yes	Yes	Yes	Yes	Yes	Yes
Veber	Yes	Yes	No	Yes	Yes	No
Egan	Yes	No	Yes	Yes	Yes	Yes
Muegge	Yes	No	No	Yes	Yes	No
Ghose	Yes	Yes	Yes	Yes	Yes	Yes

**Table 6:** Drug-like physicochemical attributes of drug candidates.

Rules	Compounds					
	3a	3b	3c	4a	4b	4c
Mol. Formula	C_13_H_10_F_3_N_3_O_2_S	C_12_H_10_N_4_O_4_S	C_14_H_9_F_6_N_3_O_2_S	C_13_H_8_F_3_N_3_OS	C_12_H_8_N_4_O_3_S	C_14_H_7_F_6_N_3_OS
Mol. Mass (g/mol)	329.20	306.3	397.30	311.28	288.28	379.28
Heavy Atoms	22	21	26	21	20	25
Aromatic heavy atoms	11	11	11	16	16	16
nHD	3	3	3	1	1	1
nHA	5	4	8	5	4	8
nRot	7	7	8	3	3	4
TPSA (Å_2_)	98.39	144.21	98.39	78.84	124.66	78.84
Molar refractivity (cm3/mol)	76.13	79.95	81.13	71.45	75.27	76.45
Fraction of Sp^3^ carbon	0.08	00	0.14	0.08	0.00	0.14
ESOL class	Soluble	Soluble	Moderately soluble	Moderately soluble	Soluble	Moderately soluble
log P	2.98	1.81	3.94	2.92	3.09	5.22

### Physicochemical attributes

The drug like physicochemical properties of synthesized compounds are displayed in Tables 5 and 6, predicted using available web resources. Compound 3b breach Egan (TPSA ≤ 131.6Å or log *p* ≤ 5.88) with evaluated TPSA of 144.21, and Muegge rule (200 ≤ MW ≤ 500, HBA ≤ 10, HBD ≤ 5, nRB ≤ 15, #rings ≤ 7, # C ≥ 4, # Hetro atom ≥ 1, TPSA), whereas 3c and 4c breach Veber rule (nRB ≤ 10 and polar surface area ≤ 140) and Muegge rule. The expected values suggest that the better oral bioavailability and systemic distribution of compounds are linked to their structural and chemical-physical attributes.

### BOILED-egg evaluation

The BOILED-Egg model assesses the oral bioavailability and BBB permeability of compounds^73^. The proposed theory suggests that the yellow yolk indicates the ability to cross the BBB, whereas the white component represents the likelihood of GI absorption. The investigation demonstrated that none of the compounds are able cross the BBB and most of them are successfully absorbed by the gastrointestinal GI tract as shown in Supplementary Figure S-24. The results indicate that the synthesized compounds have potential biological activity.

### ADMET studies

Many promising drug candidates with undesirable pharmacokinetic profiles fail to proceed to clinical trials. In silico techniques aim to provide an alternative strategy in the preliminary phase of pharmaceutical development, facilitating the evaluation of a ligand’s basic pharmacokinetic parameters and minimizing labor and expenses. The pharmacokinetic and toxicity profiles of the introduced compounds are presented in Supplementary Table S-2. They were evaluated to determine the predicted metrics of the absorption parameters using freely accessible web servers. These compounds have low buffer and water solubility as shown in Table 6. The human intestinal system was a good absorption site, ranging from 74% to 86% human intestinal absorption rate and most of the compounds showed high in-silico intestinal permeability at Caco-2 was in the range of 0.99–1.4 log Papp in 10 cm/s. The predicted skin permeability was − 2.8 to −3.11 log Kp cm/h indicating low dermal permeability. These compounds are P-glycoproteins substrate and inhibited P-glycoproteins I and II. The volume of distribution in humans for screened derivatives was below 0.05 logL/kg, displaying high distribution in tissues. The permeability values of CNS (logPS) and BBB (logBB) were considerably low, implying that these substances are unlikely to accumulate significantly in the brain and the central nervous system.

The proposed ligands exhibited distinct ADME profiles, relative to their response of interactions with multiple CYP450 enzymes, and biopharmaceutical descriptors and are non-substrates of CYP4502D6 and 3A4 whereas some of them are non-inhibitors for CYP450 2D6, and 3A4. Ligand 4c showed CYP450 1A2 and 2C19 and 3c CYP450 1A2 and 3A4 inhibition indicating possible interactions between drugs. High CYP inhibitory promiscuity was associated with an increased probability of interactions with various CYP450 enzymes.

According to the GHS (globally harmonized system), all synthesized derivatives do not fall in the GHS acute toxicity category and far above the class 5 toxicity (LD50300–2000 mg/kg). pkCSM was used to calculate the rats’ acute (mg/kg) and chronic (log mg/kg/day) oral toxicity doses. Flathead minnow fish (logmM) and T. pyriformis (logµg/L) do not exhibit significant acute oral toxicity from these compounds, suggesting minimal environmental toxicity. Moreover, there is a decreased likelihood of carcinogenicity and skin-sensitization, and the derivatives are non-inhibitors of hERG-I and hERG-II and non-hepatotoxic. However, the toxicological profile of the derivatives can be further validated through experimental cytotoxicity evaluation.

### DFT studies

**Fig. 14:**
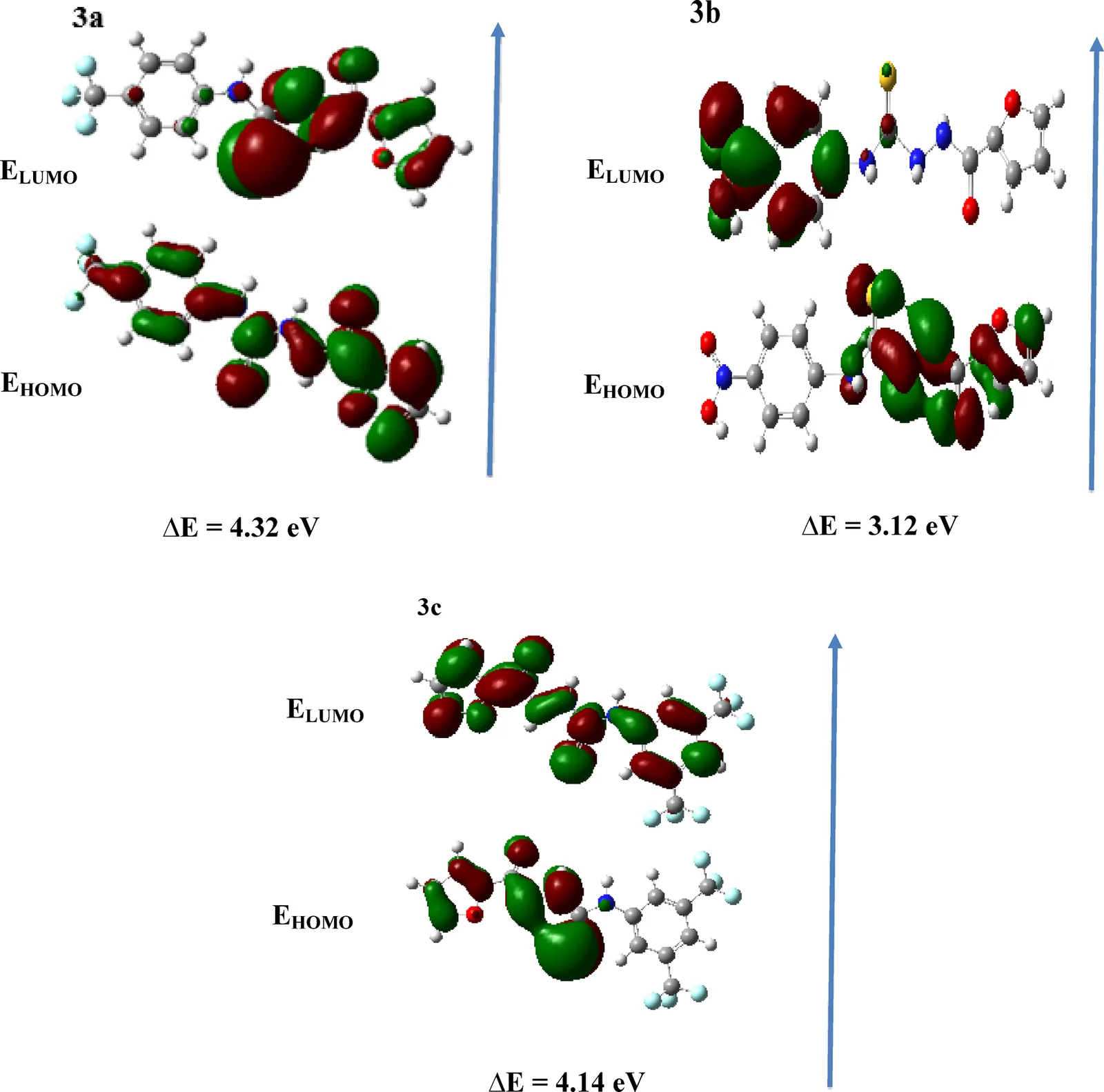
Graphical illustration of FMOs orbitals of 3a, 3b and 3c displayed their energy gaps.

The molecular geometry and substituted features significantly influence the chemical reactivity and electronic properties of synthesized derivatives. Frontier molecular orbitals (FMOs) contributes significantly in predicting molecular reactivity and stability of compounds where HOMO and LUMO orbitals define efficient electron transfer at intra- and intermolecular level. The occupied lowest energy LUMO behave as an electron acceptor and HOMO with highest energy level and electron occupancy as electron donor. The greater HOMO-LUMO energy gap (ΔE) indicates less reactivity and kinetically more stable as compared to electronic system with smaller energy gap, facilitates charge transmission, resulting in more polarized and soft species^74^. Figure 14 displayed illustration of the HOMO and LUMO orbitals of optimized structures of 3a, 3b and 3c.

The electronic indices such as HOMO energy, LUMO energy, gap between HOMO and LUMO energies, polarizability (α), dipole moment, optimization energy and reactivity parameters including electrophilicity index (ω), electronegativity (X), softness (S), hardness (η), chemical potential (µ), and charge transfer capability (ΔN max) of 3a, 3b and 3c displayed in Table 7 provide theoretical support for the observed reaction behavior. The values of E_HOMO_ and E_LUMO_ for compounds 3a-3c were − 6.476, −5.632, −6.750 eV and − 2.149, −2.503, −2.332 eV, whereas energy gap analysis is depicted as 4.326, 3.1293, and 4.418. The findings are in agreement with the literature suggested the resonance and inductive effects are preceded by para-substituted electron-withdrawing groups and, stabilize the orbitals, causing variable HOMO-LUMO gap, depending on charge delocalization^75^. The compound 3b, having nitro substituent, exhibited the lowest bandgap, more reactivity, and softness. Lower HOMO-LUMO energy gaps favor electron transmission and facilitate catalytic reaction progression. The final products exhibiting relatively larger energy gaps are often associated with enhanced electronic stabilization after formation, indicating reduced chemical reactivity and improved product stability.

The HOMO orbital distribution was evaluated to reveal an electron-donating center, and observed higher localization of HOMO density on sulfur. Significant sulfur polarizability and lone pair donor capability suggest their involvement as nucleophilic and coordination sites, and support their possible participation in the catalytic activation process. Mulliken charges analysis of 3a, 3b, and 3c showed an even charge distribution on hydrogen. Sulfur, nitrogen, and oxygen have strong negative charges, while most carbons are negatively charged due to resonance effects. These findings of uneven electron density critically affect the molecular reactivity, geometry, and interaction^76^.

The correlation between global reactivity parameters, i.e., ionization potential and electron affinity, indicates the ability an electron donor and acceptor, well explained by Nechaev in 2021^77^. Higher IP 6.75 eV of 3c showed strong molecular stability and required high energy to polarize as compared to 3a and 3b. The hardness and reactivity inverse relationship was also assessed, and found that 3b has lowest η value and corresponds to good chemical reactivity.

**Table 7:** Global reactivity parameters (GRPs), optimization energies, and electronic descriptors of 3a, 3b, and 3c.

Descriptors	Compounds		
	3a	3b	3c
Optimization energy (Hartree)	−1513.7	−1373.93	−1850.72
E_HOMO_ (a.u)	−0.238	−0.207	−0.24807
E_LUMO_ (a.u)	−0.079	−0.092	−0.0857
E_HOMO_ (eV)	−6.47628	−5.63273	−6.750307
E_LUMO_ (eV)	−2.14969	−2.50344	−2.332008
∆E (eV)	4.326597	3.1293	4.4182988
Ionization Potential (eV)	6.476289	5.632739	6.7503072
Electron Affinity (eV)	2.149693	2.50344	2.3320084
Electronegativity (eV)	4.312991	4.068089	4.5411578
Hardnes η (eV)	2.163298	1.56465	2.2091494
Softness S (eV^− 1^)	0.462257	0.639121	0.4526629
Chemical potential µ (eV)	−4.31299	−4.06808	−4.541157
Electrophilicity (eV)	4.299428	5.288516	4.6674331
ΔN max	1.993711	2.6	2.0556137

## Conclusion

The findings of this work established the synthesis of a cobalt-supported catalyst featuring a porous zeolite framework and evaluated in the nucleophilic condensation reaction for the fabrication of furan-based thiosemicarbazides and 1,2,4 triazole-3-thione derivatives in a green synthesis strategy with high product yield in a shorter time, showed reusability for at least five cycles with insignificant reduction in activity. Compound 4c revealed prominent activity in initial anticancer screening, and showed the strong binding affinities with p21 and p53 proteins outperforming the reference drug erlotinib. The developed Co-Zeolite 4 A catalyst corroborates its potential as a sustainable alternative for catalytic synthesis of 1,2,4 triazole whereas, future studies could be focused on expanding substrate applicability, the detailed mechanistic insights and in vivo validation.

## Supplementary Information

Below is the link to the electronic supplementary material.

Supplementary Material 1

## Data Availability

Data is provided within the manuscript or supplementary information files.
