# Supplementary material for: Facile Cobalt-supported Zeolite 4 A as ecofriendly catalyst to prepare 2-Furyl- hydrazine-1-carbothioamides and 1,2,4 Triazole-3-thiones with anticancer investigation
Source: Sci Rep. 2026 Jun 19;16:28150. doi: 10.1038/s41598-026-55831-z (PMC13554246; doi:10.1038/s41598-026-55831-z)
Supplement: Supplementary file 1 [file 41598_2026_55831_MOESM1_ESM.docx]

# Supporting Information for Facile Cobalt-Supported Zeolite 4A as Ecofriendly Catalyst to prepare 2-Furyl- hydrazine-1-carbothioamides and 1,2,4 Triazole-3-thiones with Anticancer Investigation

Asiya Farheen ^a, b, c^, Asia Naz Awan ^a, b^ ^[1]^, Abdul Hameed ^d^, Syed Fakhar Alam ^e, f^

*^a^ Research Institute of Pharmaceutical Sciences, University of Karachi, Karachi 75270, Pakistan*

*^b^ Department of Pharmaceutical Chemistry, Faculty of Pharmacy and Pharmaceutical Sciences, University of Karachi, Karachi 75270, Pakistan*

*^c^ Baqai Institute of Pharmaceutical Sciences, Baqai Medical University, 74600, Karachi, Pakistan.*

*^d^Department of Chemistry, University of Sahiwal, Sahiwal 57000, Pakistan*

*^e^ HEJ Institute of Chemistry, ICCBS, University of Karachi, Karachi 75250, Pakistan,*

*^f^ Graduate* *School of Energy Science and Technology, Chungnam National University, Daejeon, South Korea.*

*E-mail:asia.naz@uok.edu.pk (Asia Naz Awan)

**Catalyst characterization**

Figure S-1 BET isotherm data for Co-Zeolite 4A

Figure S-2 BET isotherm data for Zeolite 4A

Table S-1: - X-ray diffraction data of Co-Zeolite 4A:

| **No. of peaks** | **Miller indices (h k l)** | **Bragg’s angle (2Ө)** | **FWHM (deg)** | **Crystallite size (A°)** | **d-spacing (A°)** |
| --- | --- | --- | --- | --- | --- |
| **1** | 2 0 0 | 7.29 | 0.11 | 702.79 | 12.12 |
| **2** | 2 2 0 | 10.29 | 0.10 | 768.18 | 8.52 |
| **3** | 2 2 2 | 12.58 | 0.10 | 753.97 | 7.07 |
| **4** | 4 2 2 | 17.79 | 0.10 | 739.81 | 5.0 |
| **5** | 6 0 0 | 21.81 | 0.12 | 633.89 | 4.06 |
| **6** | 6 2 2 | 24.14 | 0.13 | 597.53 | 3.68 |
| **7** | 6 4 2 | 27.28 | 0.13 | 558.05 | 3.26 |
| **8** | 6 4 0 | 26.27 | 0.12 | 606.39 | 3.39 |
| **9** | 6 4 4 | 30.11 | 0.14 | 518.58 | 2.96 |
| **10** | 8 5 0 | 34.36 | 0.15 | 493.57 | 2.6 |
| **11** | 8 8 1 | 41.72 | 0.16 | 439.54 | 2.16 |
| **12** | 1 4 2 1 | 52.83 | 0.20 | 354.8 | 1.73 |

**Characterization of synthesized compounds**

Figure S-3 ^1^H-NMR spectrum of 3a

Figure S-4 ^13^C-NMR spectrum of 3a

Figure S-5 EI-MS spectrum of 3a

Figure S-6 ^1^H-NMR of 3b (MeOD)

Figure S-7 ^1^H-NMR spectrum of 3b (DMSO)

Figure S-8 EI-MS spectrum of 3b

Figure S-9 ^1^H-NMR spectrum of 3c (MeOD)

Figure S-10 ^13^C-NMR spectrum of 3c (MeOD)

Figure S-11 EI-MS spectrum of 3c (MeOD)

Figure S- 12 ^1^H-NMR spectrum of 4a (MeOD)

Figure S- 12 ^13^C-NMR spectrum of 4a (MeOD)

Figure S- 13 EI-MS spectrum of 4a

Figure S- 14 ^1^H-NMR spectrum of 4b (MeOD)

Figure S-15 EI-MS spectrum of 4b (MeOD)

Figure S-16 ^1^H-NMR spectrum of 4c (MeOD)

Figure S-17 ^13^C-NMR spectrum of 4c (MeOD)

Figure S-18 ^13^C-NMR spectrum of 4c (MeOD)

Figure S-19 FT-IR spectrum of 3a

Figure S-20 FT-IR spectrum of 3b

Figure S-21 FT-IR spectrum of 3c

Figure S-22 FT-IR spectrum of 4a

Figure S-23 FT-IR spectrum of 4c

**Table S-2: Pharmacokinetic and toxicity profile**

| **Compounds** | **Pharmacokinetics** | **Toxicity properties** | **Medicinal Chemistry** |
| --- | --- | --- | --- |
| 3a | GI absorption: High  BBB permeant: No  Caco2 permeability: -0.01  P-gp substrate: Yes  P-gp I inhibitor: No  P-gp I inhibitor: No  CYP1A2 inhibitor: Yes  CYP2C19 inhibitor: No  CYP2C9 inhibitor: No  CYP2D6 inhibitor: No  CYP3A4 inhibitor: No  Skin permeability -3.117 cm/s  Bioavailability score: 0.55 | AMES toxicity: No  Max. tolerated dose (human): 0.846  LD50: 3.43  LOAEL: 1.25  Hepatotoxicity: No  Skin sensitization: No  T.pyriformis toxicity: 1.583  Minnow toxicity: 2.219 | PAINS: 0 alert  Brenk: 1 alert: thiocarbonyl_group  Leadlikeness: Yes  Synthetic accessibility: 2.75 |
| 3b | GI absorption: High  BBB permeant: No  Caco2 permeability: -0.01  P-gp substrate: Yes  P-gp I inhibitor: No  P-gp I inhibitor: No  CYP1A2 inhibitor: Yes  CYP2C19 inhibitor: No  CYP2C9 inhibitor: No  CYP2D6 inhibitor: No  CYP3A4 inhibitor: No  Skin permeability: -2.884 cm/s  Bioavailability score: 0.55 | AMES toxicity: Yes  Max. tolerated dose (human): 1.039  LD50: 2.55  LOAEL: 1.687  Hepatotoxicity: No  Skin sensitization: No  T.pyriformis toxicity: 1.139  Minnow toxicity: 1.904 | PAINS: 0 alert  Brenk: 2 alert: nitro group, thiocarbonyl group  Leadlikeness: Yes  Synthetic accessibility: 2.79 |
| 3c | GI absorption: High  BBB permeant: No  Caco2 permeability: 0.998  P-gp substrate: Yes  P-gp I inhibitor: No  P-gp I inhibitor: No  CYP1A2 inhibitor: Yes  CYP2C19 inhibitor: No  CYP2C9 inhibitor: No  CYP2D6 inhibitor: No  CYP3A4 inhibitor: Yes  Skin permeability: -3.002 cm/s  Bioavailability score: 0.55 | AMES toxicity: No  Max. tolerated dose (human): 0.61  LD50: 3.59  LOAEL: 0.81  Hepatotoxicity: Yes  Skin sensitization: No  T.pyriformis toxicity: 1.196  Minnow toxicity: 2.10 | PAINS: 0 alert  Brenk: 1 alert: thiocarbonyl_group  Leadlikeness: 3 violations  Synthetic accessibility: 2.97 |
| 4a | GI absorption: High  BBB permeant: No  Caco2 permeability: 0.995  P-gp substrate: Yes  P-gp I inhibitor: No  P-gp I inhibitor: No  CYP1A2 inhibitor: Yes  CYP2C19 inhibitor: No  CYP2C9 inhibitor: No  CYP2D6 inhibitor: No  CYP3A4 inhibitor: No  Skin permeability: -3.11 cm/s  Bioavailability score: 0.55 | AMES toxicity: No  Max. tolerated dose (human): 0.846  LD50: 3.34  LOAEL: 1.252  Hepatotoxicity: No  Skin sensitization: No  T.pyriformis toxicity: 1.58  Minnow toxicity: 2.219 | PAINS: 0 alert  Brenk: 1 alert: thiocarbonyl_group  Leadlikeness: Yes  Synthetic accessibility: 2.57 |
| 4b | GI absorption: High  BBB permeant: No  Caco2 permeability: 0.997  P-gp substrate: Yes  P-gp I inhibitor: No  P-gp I inhibitor: No  CYP1A2 inhibitor: Yes  CYP2C19 inhibitor: No  CYP2C9 inhibitor: No  CYP2D6 inhibitor: No  CYP3A4 inhibitor: No  Skin permeability: -2.88 cm/s  Bioavailability score: 0.55 | AMES toxicity: No  Max. tolerated dose (human): 1.03  LD50: 2.7  LOAEL: 1.6  Hepatotoxicity: No  Skin sensitization: No  T.pyriformis toxicity: 0.86  Minnow toxicity: 1.90 | PAINS: 0 alert  Brenk: 2 alerts: nitro group, thiocarbonyl_group  Leadlikeness: No  Synthetic accessibility: 2.64 |
| 4c | GI absorption: Low  BBB permeant: No  Caco2 permeability: 1.484  P-gp substrate: Yes  P-gp I inhibitor: Yes  P-gp I inhibitor: No  CYP1A2 inhibitor: Yes  CYP2C19 inhibitor: Yes  CYP2C9 inhibitor: No  CYP2D6 inhibitor: No  CYP3A4 inhibitor: No  Skin permeability: -2.95 cm/s  Bioavailability score: 0.55 | AMES toxicity: No  Max. tolerated dose (human): 0.657  LD50: 3.81  LOAEL: 0.522  Hepatotoxicity: Yes  Skin sensitization: No  T.pyriformis toxicity: 1.025  Minnow toxicity: 2.11 | PAINS: 0 alert  Brenk: 1 alert: thiocarbonyl_group  Leadlikeness: No  Synthetic accessibility: 2.79 |

|  3a 3b |  |  |  |  |  |  |  |  |  |  |   3c | | | | |
| --- | --- | --- | --- | --- | --- | --- | --- | --- | --- | --- | --- | --- | --- | --- | --- |
|  |  |  |  |  |  |  |  |  |  |  |  |  |  |  |  |
|  |  |  |  |  |  |  |  |  |  |  |  |  |  |  |  |
|  |  |  |  |  |  |  |  |  |  |  |  |  |  |  |  |
|  |  |  |  |  |  |  |  |  |  |  |  |  |  |  |  |
|  |  |  |  |  |  |  |  |  |  |  |  |  |  |  |  |
|  |  |  |  |  |  |  |  |  |  |  |  |  |  |  |  |
|  |  |  |  |  |  |  |  |  |  |  |  |  |  |  |  |
|  |  |  |  |  |  |  |  |  |  |  |  |  |  |  |  |
|  |  |  |  |  |  |  |  |  |  |  |  |  |  |  |  |
|  |  |  |  |  |  |  |  |  |  |  |  |  |  |  |  |
|  |  |  |  |  |  |  |  |  |  |  |  |  |  |  |  |
|  |  |  |  |  |  |  |  |  |  |  |  |  |  |  |  |
|  |  |  |  |  |  |  |  |  |  |  |  |  |  |  |  |
|  4a 4b |  |  |  |  |  |  |  |  |  |  | 4c |  |  |  |  |
|  |  |  |  |  |  |  |  |  |  |  |  | | | | |
|  |  |  |  |  |  |  |  |  |  |  |  |  |  |  |  |
|  |  |  |  |  |  |  |  |  |  |  |  |  |  |  |  |
|  |  |  |  |  |  |  |  |  |  |  |  |  |  |  |  |
|  |  |  |  |  |  |  |  |  |  |  |  |  |  |  |  |
|  |  |  |  |  |  |  |  |  |  |  |  |  |  |  |  |
|  |  |  |  |  |  |  |  |  |  |  |  |  |  |  |  |
|  |  |  |  |  |  |  |  |  |  |  |  |  |  |  |  |
|  |  |  |  |  |  |  |  |  |  |  |  |  |  |  |  |
|  |  |  |  |  |  |  |  |  |  |  |  |  |  |  |  |
|  |  |  |  |  |  |  |  |  |  |  |  |  |  |  |  |
|  |  |  |  |  |  |  |  |  |  |  |  |  |  |  |  |
|  |  |  |  |  |  |  |  |  |  |  |  |  |  |  |  |
|  |  |  |  |  |  |  |  |  |  |  |  |  |  |  |  |
|  |  |  |  |  |  |  |  |  |  |  |  |  |  |  |  |

Figure S-24 ADME profile of compounds 3a-c and 4a-c

Figure S-25 C) 2D Binding models of Erlotinib in the active sites of p21 (PDB ID 2CDZ) D) 2D Binding models of Erlotinib in the active sites of p53 (PDB ID 5G4M).

1. Correspondence: Asia Naz Awan ^a, b^
